# Circular RNAs: Emerging Role in Cancer Diagnostics and Therapeutics

**DOI:** 10.3389/fmolb.2020.577938

**Published:** 2020-10-28

**Authors:** Anuva Rajappa, Sucharita Banerjee, Vivek Sharma, Piyush Khandelia

**Affiliations:** Department of Biological Sciences, Birla Institute of Technology and Science, Pilani - Hyderabad Campus, Hyderabad, India

**Keywords:** circRNAs, cancer, biomarkers, diagnostics, therapeutics

## Abstract

Circular RNAs (circRNAs) are rapidly coming to the fore as major regulators of gene expression and cellular functions. They elicit their influence *via* a plethora of diverse molecular mechanisms. It is not surprising that aberrant circRNA expression is common in cancers and they have been implicated in multiple aspects of cancer pathophysiology such as apoptosis, invasion, migration, and proliferation. We summarize the emerging role of circRNAs as biomarkers and therapeutic targets in cancer.

## Introduction

Our understanding of the human transcriptome has increased significantly by the discovery and understanding of the role of regulatory non-coding RNAs in physiology and diseases such as cancer (Vo et al., 2019). Among the non-coding regulatory transcripts, circRNAs have attracted intense research scrutiny in recent years (Chen, 2020). CircRNAs are single-stranded covalently closed continuous loop structures lacking free ends and a polyadenylate tail (Li X. et al., 2018b; Kristensen et al., 2019; Chen, 2020). Close to one-fifth of active genes in the human genome can potentially give rise to circRNAs (Salzman et al., 2012; Li X. et al., 2018b; Kristensen et al., 2019; Chen, 2020). CircRNAs are composed of exonic and/or intronic sequences and are primarily generated by back-splicing, a non-canonical alternative RNA splicing event mediated by the spliceosome and regulated by a combination of *cis*-elements and *trans*-factors (Chen and Yang, 2015; Li X. et al., 2018b; Kristensen et al., 2019; Chen, 2020). Due to the absence of free ends circRNAs are not susceptible to destruction by RNA degradation machinery and are more stable than linear RNAs (Lasda et al., 2014; Wang et al., 2015; Zhang Y. et al., 2016). The majority but not all circRNAs are non-coding and exhibit their biological functions by sequestration of miRNAs/proteins. Some circRNAs regulate transcription, splicing and may also be translated to polypeptides. CircRNAs are involved in the regulation of cancer hallmarks such as self-sustenance in growth signals, proliferation, angiogenesis, resistance to apoptosis, unlimited replicative potential, and metastasis (Shi, 2017; Bach et al., 2019; Vo et al., 2019). Here we summarize and catalog the advances in the use of circRNAs as biomarkers for cancer diagnosis and as therapeutic targets.

## Biogenesis of Circular RNAs

**Figure 1:**
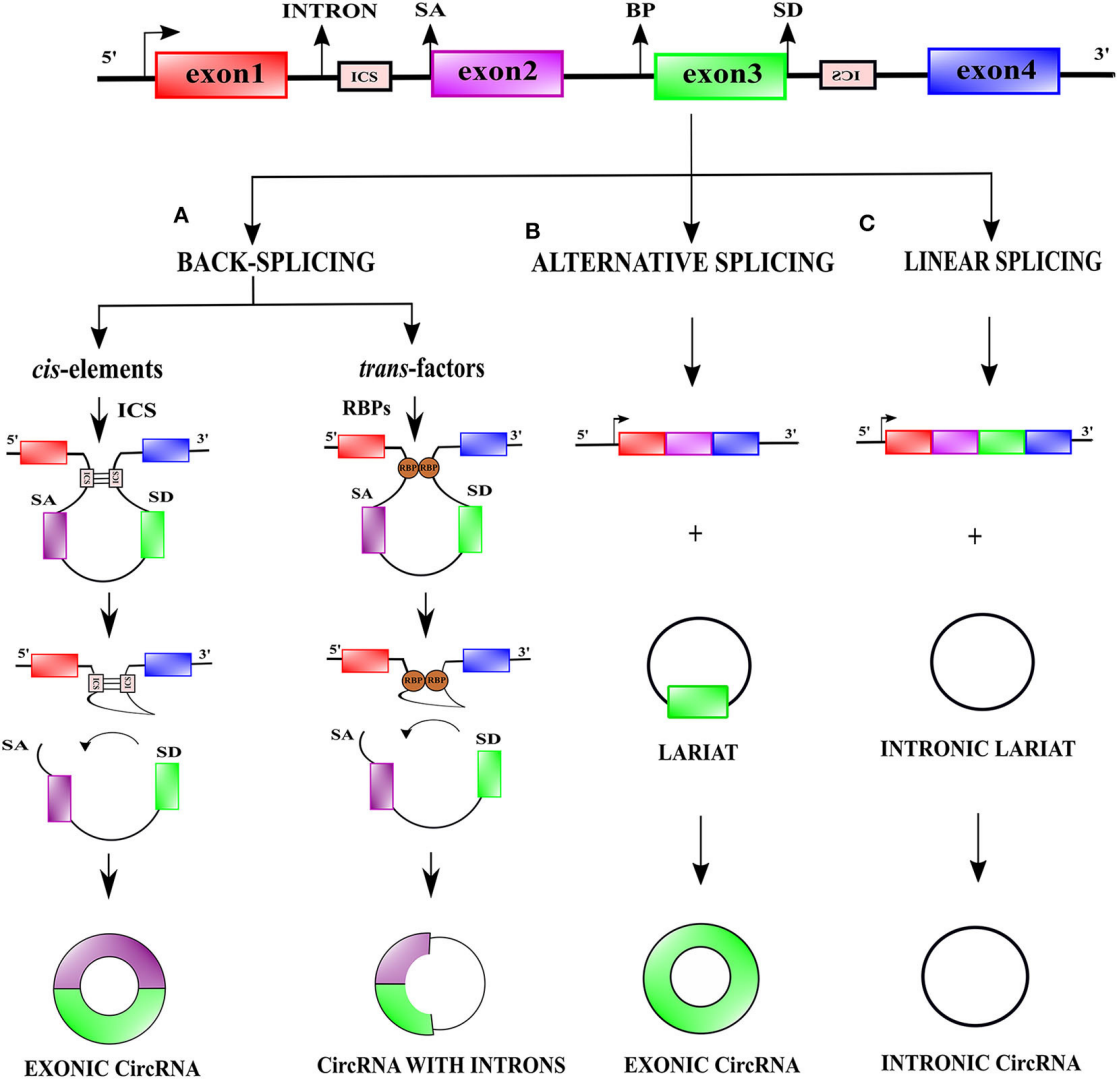
Biogenesis of circular RNAs. **(A)** During transcription, linear and back-splicing of exons rival each other. Back-splicing is facilitated by long flanking introns, cis-elements i.e., intronic complementary elements (ICS), and trans-factors i.e., RNA-binding proteins (RBPs). To facilitate back-splicing, a downstream splice-donor (SD) site is brought in close vicinity with an upstream splice-acceptor (SA) site *via* base-pairing interactions between ICS or dimerization of RBPs. An upstream branch point (BP) nucleophilically attacks a downstream SD site, which thereafter nucleophilically attacks an upstream SA site, resulting in the creation of exonic circRNAs or exon-intron circRNAs. **(B)** Alternative splicing events like exon-skipping often generate skipped exon bearing lariat precursors, which can participate in the genesis of exonic circRNAs. **(C)** Intronic lariat precursors generated by linear canonical splicing can escape lariat debranching and serve as a source for intronic circRNAs.

In eukaryotes, the generation of a mature mRNA is a result of interaction between transcription, splicing, capping, polyadenylation, export, and degradation (Black, 2003; Moore and Proudfoot, 2009; Nilsen and Graveley, 2010). CircRNAs are formed by a specialized non-conventional alternative splicing referred to as back-splicing (Zhang X. O. et al., 2016). In contrast to the classical canonical splicing, during back-splicing, a downstream 5' splice-site is joined to an upstream 3' splice-site across a single or multiple exons leading to the formation of circRNA species (Figure 1A) (You et al., 2015; Li X. et al., 2018b; Kristensen et al., 2019; Chen, 2020).

Two models have been proposed to explain the coupling of back-splicing to canonical splicing for circRNAs biogenesis (i) “exon-skipping” or “lariat-intermediate” model and (ii) “direct back-splicing” model (Lasda et al., 2014). In the “lariat intermediate” model, canonical splicing occurs first and generates an intronless linear RNA, and an intron lariat bearing skipped exons which eventually undergoes back-splicing (Zaphiropoulos, 1996; Kelly et al., 2015) (Figures 1B,C). In the “direct back-splicing” model back-splicing occurs first leading to the formation of a circRNA followed by the creation of a linear RNA (Li Y. et al., 2017) (Figure 1A). Based on their origin, circRNAs fall into three major classes, exonic, intronic, and exon-intron circRNAs. Except for splice-sites, no particular sequences are necessary for circularization, however, a median exonic length is required for back-splicing involving either single or multiple exons (Ashwal-fluss et al., 2014; Zhang et al., 2014).

CircRNA synthesis by back-splicing occurs both co-transcriptionally and post-transcriptionally and is favored by a high rate of transcription elongation (Ashwal-fluss et al., 2014; Zhang Y. et al., 2016; Vo et al., 2019). The ligation of a downstream 5′ splice-site with an upstream 3′ splice-site during back-splicing is not favored sterically leading to lower efficiency of back-splicing as opposed to conventional linear splicing (Jeck et al., 2013; Zhang Y. et al., 2016). Interestingly, alternative back-splicing events can also occur and generate multiple circRNA isoforms (Gao et al., 2016; Zhang X. O. et al., 2016). Just like linear RNAs, circRNAs too are subjected to widespread reversible modification, in particular N6-methyladenosine (m6A) modification, which may influence their cellular fate (Zhou et al., 2017).

### Role of Cis-Elements and Trans-Factors in Circular RNA Formation

CircRNA formation by back-splicing is facilitated by *cis*-elements such as intronic complementary sequences (ICS), flanking circRNA forming exons and *trans*-factors like RNA-binding proteins (RBPs) (Figure 1A) (Jeck et al., 2013; Ashwal-fluss et al., 2014; Liang and Wilusz, 2014; Zhang et al., 2014). ICS facilitate RNA pairing, by bringing distal splice-sites close to each other, which promotes circularization (Jeck et al., 2013). In humans, both complementary inverted-repeat *Alu* elements located in introns, as well as non-repetitive complementary sequences in introns, promote RNA pairing and subsequent back-splicing (Jeck et al., 2013; Liang and Wilusz, 2014; Zhang et al., 2014; Starke et al., 2015). *Trans*-factors contribute to circRNA biogenesis by modulating back-splicing: (i) by directly bridging distal splice-sites (ii) by binding to ICS. Some examples of *trans*-factors are RBPs such as Quaking (QKI), Heterogeneous-nuclear ribonucleoprotein L (HNRNPL), and RNA-binding motif protein 20 (RBM20) (Figure 1A) (Conn et al., 2015; Errichelli et al., 2017). RBPs which bind to ICS and regulate circRNA biogenesis bear double-stranded RNA-binding domains (dsRBDs) and can stabilize or destabilize the base-pairing between ICSs to promote or prevent back-splicing. The dsRBDs which promote back-splicing are nuclear factor 90 (NF90) and nuclear factor (NF110) (Patiño et al., 2015; Li et al., 2017) whereas dsRBDs which prevent back-splicing include DExH-Box Helicase 9 (DHX9) and adenosine deaminase 1 acting on RNA (ADAR1) (Ivanov et al., 2015; Aktaş et al., 2017).

## Mechanism of Action of Circular RNAs

### Circular RNAs as miRNA Sponges

**Figure 2:**
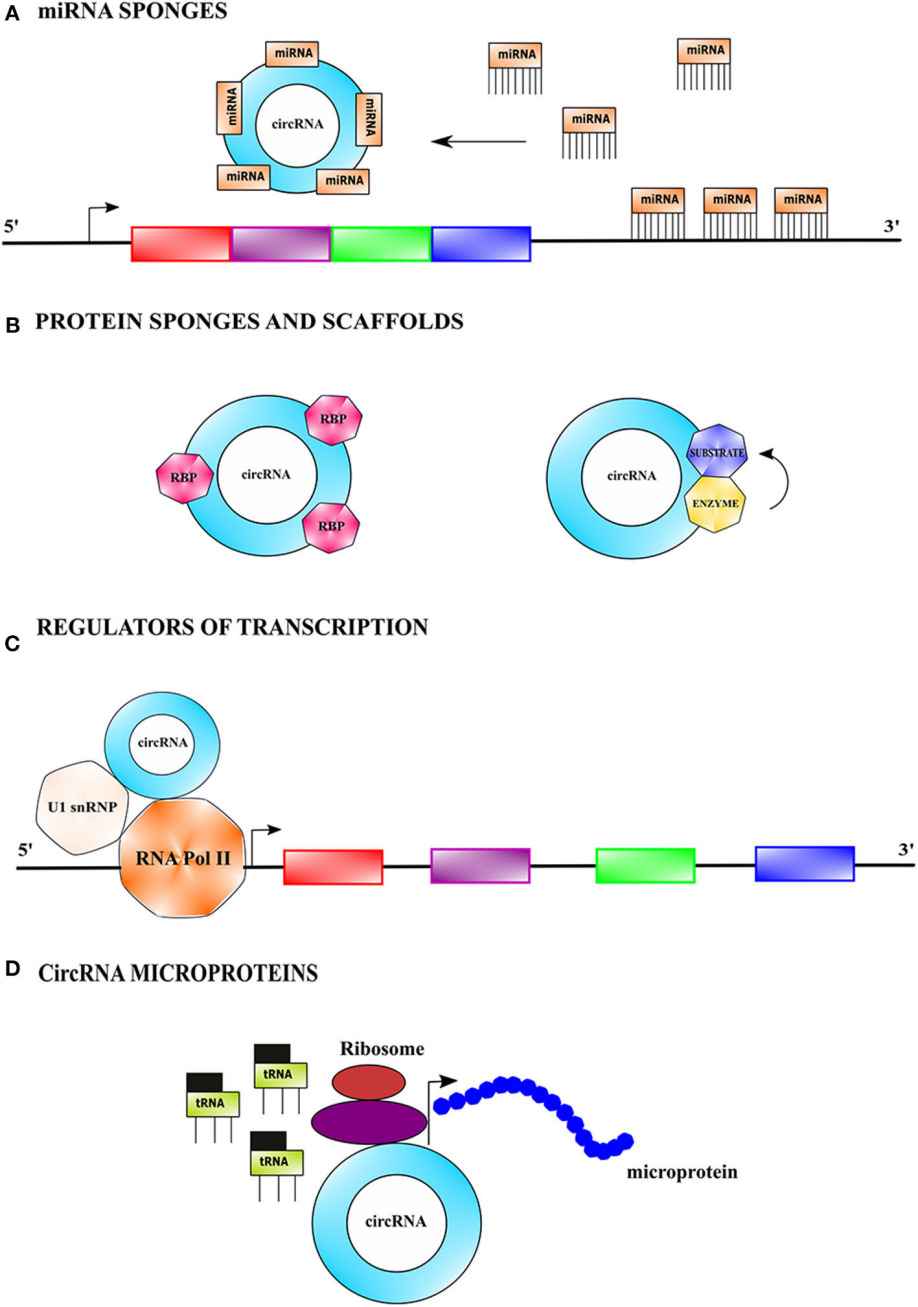
Mechanistic action of circular RNAs. **(A)** CircRNAs can serve as miRNA sponges by competitively binding to miRNA(s) *via* base-pairing interactions, causing stabilization of target transcript(s) of the sequestered miRNA(s), and thus making it more available for translation. **(B)** CircRNAs can sponges protein by binding to them and thus indirectly regulating their functions. CircRNAs can also function as protein scaffolds by facilitating colocalization of an enzyme and its substrate to influence the kinetics of catalysis. **(C)** CircRNAs can modulate transcription by binding to RNA polymerase II complex bearing the U1 small nuclear ribonucleoprotein among other proteins and augment the function of certain proteins of the complex. **(D)** CircRNAs bearing internal ribosome entry site (IRES) elements and initiation codons can initiate translation in a cap-independent fashion and generate short polypeptides referred to as microproteins.

CircRNAs competitively bind and sponge miRNAs leading to the stabilization of their target transcripts. They can have single or multiple binding sites for single or several miRNAs (Figure 2A). For instance, the expression of miR-7 target genes is regulated by *CDR1as*, which harbors >70 conserved binding sites for miR-7 (Hansen et al., 2013). Some circRNAs acting as miRNA sponges have oncogenic and tumor-suppressive properties (Kristensen et al., 2019). For example, *circCCDC66* binds two miRNAs, miR-33b and miR-93, and promotes tumorigenesis in colorectal cancer by upregulation of c-MYC (Hsiao et al., 2017).

### Circular RNAs as Protein Sponges

Interestingly, circRNAs can also bind to proteins and prevent their activity (Figure 2B) (Ashwal-fluss et al., 2014). For example, *circPABPN1* binds to the Hu-antigen R (HuR) and prevents its binding to the cognate linear mRNA resulting in its reduced translation (Abdelmohsen et al., 2017). Certain circRNAs bind to multiple proteins and hold them together as a scaffold to facilitate their interaction. For example, *circAMOTL1* simultaneously binds to both AKT1 and PDK1 in cardiac tissue serving as a scaffold to facilitate the phosphorylation of AKT1 (protein kinase B) by PDK1 (3-phosphoinositide-dependent protein kinase 1) (Zeng et al., 2017).

### Circular RNAs as Regulators of Transcription and Splicing

Nuclear circRNAs also modulate transcription and splicing (Figure 2C). For example, the intronic *ci-ankrd52* assembles at the transcription sites of its cognate gene and positively regulates RNA polymerase II driven transcription (Yang Y. et al., 2017). *CircRNAs EIF3J* and *PAIP2* interact and form complexes with U1snRNP, which in turn interact with RNA polymerase II at the promoters of the parental genes, leading to transcriptional enhancement (Li Z. et al., 2015). Some circRNAs also regulate alternative splicing e.g., *circ-UBR5* modulates RNA splicing by binding to splicing regulators such as QKI, NOVA1, and U1snRNA (Qin et al., 2018; Chen, 2020).

### Functions of Circular RNAs Encoded Microproteins

Most circRNAs are noncoding but a few circRNAs have short open reading frames (ORFs) which are translated into short peptides referred to as microproteins (Figure 2D). Generally, microproteins are <100 amino acids in length and possess distinct functions as compared to the protein coded by their cognate linear mRNA (Hanada et al., 2009; Andrews and Rothnagel, 2014). CircRNAs undergo cap-independent translation facilitated by internal ribosomal entry sites (IRESs) and m6A modification in the 5'untranslated region (5'UTR) (Abe et al., 2015). Examples of microproteins encoding circRNAs include *circFBXW7, circZNF609, circMbl, circPINTexon2*, and *circSHPRH* (Motegi et al., 2006; Akhoondi et al., 2007; Yang Y. et al., 2018; Zhang M. et al., 2018; Zhang et al., 2019).

## Circular RNAs as Cancer Biomarkers

CircRNAs have several attributes that make them potential biomarkers for cancer diagnosis and prognosis. They are more stable than linear RNAs due to lack of free 5′ and 3′ ends (Memczak et al., 2013; Li Z. et al., 2015; Zhang Z. et al., 2018; Vo et al., 2019), and often display tissue and developmental stage-specific expression pattern, and can be quantitatively detected by reverse transcription followed by real-time quantitative polymerase chain reaction (RT-qPCR) (Panda and Gorospe, 2018). Moreover, altered expression of circRNAs has been frequently observed in cancer tissues and/or in plasma, and saliva from cancer patients (Memczak et al., 2013, 2015; Bahn et al., 2015; Li Z. et al., 2015; Panda and Gorospe, 2018; Zhang Z. et al., 2018; Vo et al., 2019). Li et al. first reported the presence of circRNAs in exosomes in serum of cancer patients and several cancer cell types and coined the term exo-circRNAs (Li Y. et al., 2015). The presence of exo-circRNAs in a variety of human bodily fluids that may be assessed easily without biopsy, makes exo-circRNAs a good choice for cancer diagnosis (Bai et al., 2019; Geng X. et al., 2020). Numerous circRNAs are dysregulated in cancer but few have the potential to serve as biomarkers for cancer and are summarized and cataloged in Table 1.

**Table 1:** Biological functions and roles of circular RNAs in cancer diagnostics and therapeutics.

**circRNA**	**Associated Cancer (s)**	**Regulation (Up ↑, Down↓)**	**Biological Function**	**Target (s)**	**Type of model**	**Type of cell line (s)**	**Biomarker/therapeutics**	**References**
*circ_0009910*	AML	↑	Sponges miR-20a-5p and inhibits apoptosis. Overexpressing miR-20a-5p counteracts chemoresistance *in vitro* and *in vivo* through targeting KIF26B by modulating the activities of the MAPK/ERK and cAMP/PKA signaling pathways	miR-20a-5p	Human, Mouse; *in vitro*/*in vivo*	Human cell lines – Mono-Mac-6, KG-1, AML2, AML5	+/−	Ping et al., 2019a
	GC	↑	Associated with distant metastasis and differentiation; downregulation reduces GC cell proliferation, migration and invasion. Acts as oncogene that acts by inducing the EMT. Its knockdown suppresses the migration invasion and EMT of GC cells *in vitro*.	–	Human; *in vitro*	Human cell lines – BGC823, SGC7901, AGS, MGC803, MKN45, GES1	+/+	Liu M. et al., 2018
	CML	↑	Promotes cell growth and imatinib resistance, reduces apoptosis and autophagic activation. It accelerates imatinib-resistance in cells by modulating ULK1-induced autophagy *via* targeting miR-34a-5p, providing a potential target in imatinib resistance of CML.	miR-34a-5p	Human; *in vitro*	Human cell lines – K562, K562/R	+/+	Cao et al., 2020
	OSC	↑	Sponges miR-449a which targets IL6R; prohibits cell-cycle arrest, promotes proliferation and inhibits apoptosis.	miR-449a	Human; *in vitro*	Human cell lines – MG63, Saos-2, U2OS, hFOB	+/+	Deng et al., 2018
*circ-vimentin*	AML	↑	Associated with poor overall survival (OS), leukemia-free survival and vimentin expression.	–	Human; *in vivo*	–	+/−	Yi and Lin, 2018
*circ_0075001*	AML	↑	Lowers expression of components of Toll-like receptor signaling pathway	–	Human; *in vitro*	Human cell lines – NB-4, KASUMI-1, OCI-AML5, OCI-AML3, ME-1, MV4-11, K562	+/−	Hirsch et al., 2017
*circ_0004277*	AML	↓	–	–	Human; *in vivo*	–	+/−	Li W. et al., 2017
*circ_100053*	CML	↑	Associated with clinical stage, BCR/ABL mutant status and imatinib resistance	–	Human; *in vivo*	–	+/−	Ping et al., 2019b
*circ–RPL15*	CLL	↑	Inhibits miR-146b-3p mediated suppression of the RAS/RAF1/MEK/ERK pathway	miR-146b-3p	Human; *in vitro*	Human cell lines – MEC-1 and JVM-3	+/+	Wu Z. et al., 2019
*circ-CBFB*	CLL	↑	Activates Wnt/β-catenin signaling pathway	miR-607	Human; *in vitro*	Human cell line - MEC-1	+/+	Xia L. et al., 2018
*circ_0007841*	MM	↑	Correlated with chromosomal aberrations such as gain 1q21, t (4:14), mutations in ATR and IRF4 genes; miR-199a-3p affects the multi-chemoresistance of OS *via* targeting AK4; overexpression correlates with osteolytic bone destruction in MM; overexpressed in BTZ-resistant MM cell lines	miR-199a-3p	Human; *in vitro*	Human cell lines – THP-1, KM3, U266, RPMI-8226, KM3/BTZ, 146 U266/BTZ, RPMI-8226/BTZ	+/−	Gao et al., 2019
*circ_0000190*	MM	↓	Correlates with prognosis survival rates of MM patients, inhibits MM progression *via* modulating miR-767-5p/MAPK4 pathway	miR-767-5p	Human, Mouse; *in vitro*/*in vivo*	Human cell lines – MM.1S, NCI-H929	+/+	Feng et al., 2019
	GC	↓	Associated with tumor diameter, lymphatic metastasis, distal metastasis, TNM stage and CA19-9 level	–	Human; *in vivo*	–	+/–	Chen et al., 2017
	OSC	↓	Correlated with bigger tumor size, advanced staging (IIB/III) and distant metastasis	miR-767-5p	Human, Mouse; *in vitro*/*in vivo*	Human cell lines –hFOB1.19, SAOS-2, MG63, U2OS, SJSA1, and HOS	+/–	Li et al., 2020
*circ-SMARCA5*	MM	↓	Higher expression is correlated with lower β2-MG level and less advanced ISS stage; native resistance to drugs is common due to multiple chromosomal abnormities in the pathogenesis of MM	miR-767-5p	Human; *in vitro*	Human cell lines – NCI-H929, RPMI8226, U226, OPM2, JJN3	+/+	Liu H. et al., 2019
	GC	↓	Correlates with differentiation, lymph node metastasis, vascular invasion	–	Human; *in vitro*	Human cell lines – GES-1, MGC803, MKN45, AGS, MKN74, BGC-823, SGC-7901	+/–	Cai et al., 2019
	HCC	↓	Promotes apoptosis and expression of tumor suppressor TIMP3, inhibits proliferation, invasion and metastasis; reverses tumor growth along with decreased expression of MMP9 and MMP7	miR-17-3p miR-181b-5p	Human; *in vitro*	Human cell lines – Huh7, HCCLM9, HepG2	+/+	Li Z. et al., 2019
*circRNA_101237*	MM	↑	Increases significantly in bortezomib-resistant cell lines; overexpression is associated with a poor response to chemotherapy in MM patients	–	Human; *in vitro*	Human cell lines – THP-1, MM.1S, H929, MM.1S/BTZ, H929/BTZ	+/–	Liu and Wang, 2020
	HCC	↑	Associated with tumor size, lymph node metastasis, distant metastasis and TNM stage; cisplatin resistance associated with loss of Runt- associated transcription factor 3 and upregulation of cyclophilin B	–	Human; *in vitro*	Human cell lines – HCCLM3, Hep3B, MHCC97-H, cisplatin-resistant Huh7/DDP cells	+/–	Zhou et al., 2020
*circ-APC*	BCL	↓(DLBCL)	Inhibits Wnt/β-catenin signaling	miR-888	Human, Mouse; *in vitro*/*in vivo*	Human cell lines – SUDHL-3, U2932, TMD8, OCI-Ly3, L428, GM12878	+/+	Hu et al., 2019
*CDR1as*	CRC	↑	Enhances EGFR/RAF1/MAPK pathway, induces cell growth, resistance to apoptosis and cell cycle arrest	miR-7	Human; *in vitro*	Human cell lines –HCT-116, DLD-1, NCM460, CCD841CoN	+/+	Weng et al., 2017
	HCC	↑	Promotes HCC progression by activating PI3K/AKT/mTOR pathway	miR-7	Human; *in vivo*	–	+/+	Xu et al., 2017
	GC	↑	Activates PTEN/PI3K/AKT pathway	miR-7	Human, Mouse; *in vitro*/*in vivo*	Human cell lines – MGC-803, HGC-27, GES-1	+/+	Pan et al., 2018
*circCCDC66*	CRC	↑	Promote CRC growth and metastasis by stabilizing MYC mRNA	miRNA-33b, miR-93	Human; *in vitro*/*in vivo*	Human cell lines – HCT-116, HT-29	+/–	Hsiao et al., 2017
*circ_0004585*	CRC	↑	Associated with increased patient's tumor size	–	Human; *in vivo*	–	+/–	Tian et al., 2019
*circ_0007142*	CRC	↑	Regulates proliferation and invasion of CRC; upregulation is associated with lymphatic metastasis	miR-103a-2-5p	Human; *in vitro*	Human cell lines – HCT-116, HT-29, LoVo, HCO	+/–	Zhu et al., 2019
*circHUEW1*	CRC	↑	Associated with lympho-vascular invasion, lymph node metastasis, distant metastasis, and TNM stage; affects IGF2/β-catenin signaling pathway	miR-486	Human; *in vivo, in vitro*	Human cell lines – HCT116, SW480	+/+	Chen H. Y. et al., 2020
*circ_0001178*	CRC	↑	Metastatic clinical features, advanced TNM stage and adverse prognosis; induces EMT through increasing ZEB1 expression	miR-382, miR-587 and miR-616	Human, Mouse; *in vitro*/*in vivo*	Human cell lines – NCM460 cells, CRC LoVo, SW620	+/+	Ren et al., 2020
*circ_0005075*	CRC	↑	Downregulation modulated Wnt/β-catenin pathways and reduced cell proliferation and metastasis; Knockdown suppresses EMT progression by decreasing the levels of Vimentin and N-cadherin	–	Human; *in vivo, in vitro*	Human cell lines – HCO, SW480, SW620, HT29, HCT116, SW1116, LOVO	+/+	Jin et al., 2019
	HCC	↑	Contributes to HCC proliferation, invasion, and metastasis	miR-23b-5p, miR-93-3p, miR581,miR-23a-5p	Human; *in vivo*	–	+/–	Shang et al., 2016
*circHIPK3*	CRC	↑	Promotes CRC progression,increases expression of downstream oncogenic target genes, FAK, IGF1R, EGFR, and YY1 that activate PI3K/AKT and MEK/ERK signaling pathways to promote cancer progression and drug resistance	miR-7	Human, Mouse; *in vitro*/*in vivo*	Human cell lines – FHC, HCT116, HT29, SW480, SW620, DLD1	+/+	Zeng et al., 2018
	OSC	↓	Correlates with Enneking stage and lung metastasis.	–	Human; *in vitro*	Human cell lines – SaoS2, HOS, KH-OS, MG63, 143B, U2OS	+/–	Kun-peng et al., 2018b
	BCa	↓	Reduces aggressiveness and metastasis by targeting the miR-558/heparanase axis	miR-558	Human, Mouse; *in vitro*/*in vivo*	Human cell lines –T24T, UMUC3, SV-HUC-1, HUVEC,	+/+	Li Y. et al., 2017
*circ_0001649*	CRC	↓	–	–	Human; *in vitro*	Human cell line - H116	+/–	Ji et al., 2018
	HCC	↓	Associated with tumor size, occurrence of tumor embolus; correlates with metastasis,	–	Human; *in vitro*	Human cell lines – HCC–LM3, MHCC-97L	+/–	Qin et al., 2016
	LC	↓(NSCLC)	associated with shorter OS, positive lymph node, and differentiation grade	miR-331-3p miR-338-5p	Human, Mouse; *in vitro*/*in vivo*	Human cell lines – A549, H358, H1299, H1581, 16HBE	+/–	Liu H. et al., 2018
*circITGA7*	CRC	↓(CRC)	Inactivates Ras signaling pathway; associated with tumor size, lymph metastasis, distant metastasis, and TNM stage	miR-370-3p	Human, Mouse; *in vitro*/*in vivo*	Human cell lines – SW480, RKO, Caco-2, SW620, LoVo, HCT116, DLD1, FHC	+/–	Li X. et al., 2018a
*circ_0000711*	CRC	↓	–	–	Human; *in vitro*	Human cell lines – NCM460, HCT116, COLO205, HT29	+/–	Li J. et al., 2018b
*circ_0014717*	CRC	↓	Overexpression promotes G0/G1 phase arrest, reduces growth, invasion and distal metastasis	–	Human, Mouse; *in vitro*/*in vivo*	Human cell lines – HCT116, HT29, SW480, FHC	+/–	Wang F. et al., 2018
*circFBXW7*	BC	↓(TNBC)	Inhibits tumor progression; up-regulates FBXW7 and degrades c-Myc, negatively correlated with metastasis	miR-197-3p	Human, Mouse; *in vitro*/*in vivo*	Human cell lines – MCF-10A, MCF-7, T47D, BT474, SKBR-3, MDA-MB-453, MDA-MB-468, MDAMB-231, BT549, HCC38, 4T1, MA-891	+/+	Ye F. et al., 2019
*circSEPT9*	BC	↑(TNBC)	Activates LIF/Stat3 signaling pathway, correlates with lymph node metastasis	miR-637	Human, Mouse; *in vitro*/*in vivo*	Human cell lines – MDA-MB-231, BT-549, MDA-MB-468, MDA-MB-453, SUM-159, MCF-10A	+/–	Zheng et al., 2020
*circ_0001785*	BC	↑(BC)	Associated with histological grade, TNM stage and distant metastasis	–	Human; *in vivo*	–	+/–	Yin et al., 2018
*circCDYL*	BC	↑	Regulates miR-1275-ATG7/ULK1 axis; downregulates the expression of autophagy associated genes AKT and ULK1; enhances malignant progression	miR-1275	Human, Mouse; *in vitro*/*in vivo*	Human cell lines – MDA-MB231, MCF-7	+/+	Liang et al., 2020
	MM	↑	Promotes MM growth by targeting YAP; inhibits apoptosis	miR-1180	Human, Mouse; *in vitro*/*in vivo*	Human cell lines – MM1.S, NCI-H929	+/+	Chen F. et al., 2020
*circKIF4A*	BC	↑(TNBC)	Induces TNBC cell proliferation and migration regulating the expression of KIF4A; miR-375 can sensitize resistant cells to tamoxifen and partly reverse EMT	miR-375	Human, Mouse; *in vitro*/*in vivo*	Human cell lines – MCF10A, MCF-7, T47D, BT474, KBR3, MDA-MB-453, MDA-MB-468, MDA-MB-231, BT549, HCC38	+/–	Tang et al., 2019
*circPLK1*	BC	↑(TNBC)	Promotes TNBC cell proliferation and metastasis by regulating PLK1	miR-296-5p	Human, Mouse; *in vitro*/*in vivo*	Human cell lines – MCF10A, MDAMB-468, MDA-MB-453, MDA-MB-231, HCC38, BT549	+/–	Kong Y. et al., 2019
*circHMCU*	BC	↑(BC)	Enhanced proliferation and metastasis; can modify EMT pathway, promotes mesenchymal phenotypes and inhibits epithelial phenotypes; stable and resistant to ActD treatment	let-7 family	Human, Mouse; *in vitro*/*in vivo*	Human cell lines –MDA-MB-231, MDA-MB-468, MCF7	+/–	Song et al., 2020
*circ_0068033*	BC	↓(BC)	Overexpression induces apoptosis	miR-659	Human, Mouse; *in vitro*/*in vivo*	Human cell lines – MCF10A, MCF-7, T47D, MDA-MB-468	+/–	Yuan et al., 2020
*circ_0005075*	HCC	↑	Contributes proliferation, invasion, and metastasis	miR-23b-5p, miR-93-3p, miR581 and miR-23a-5p	Human; *in vivo*	–	+/–	Shang et al., 2016
*circ_100338*	HCC	↑	Increased cell metastasis progression; regulates the MTOR signaling pathway	miR-141-3p	Human; *in vitro*	Human cell lines –Hep3B, BEL7402, MHCC97H, HCCLM6	+/+	Huang X. Y. et al., 2017
*circRHOT1*	HCC	↑	Promotes HCC progression, recruits TIP60,enhances invasion, inhibits apoptosis, and promotes metastasis	–	Human, Mouse; *in vitro*/*in vivo*	Human cell lines –Hep3B, Huh7	+/+	Wang L. et al., 2019
*circ_0091579*	HCC	↑	Associated with poor OS	–	Human; *in vivo*		+/–	Zhang C. et al., 2018
*circ-HOMER1*	HCC	↑	Increases the expression of CXCL6; associated with larger tumor size, higher TNM stage, and worse prognosis	miR-1322	Human; *in vitro*	Human cell lines – Sk-Hep-1, SMMC-7721, HCCLM3, Huh-7, HepG2 cells, L02	+/–	Zhao M. et al., 2020
*circ_0016788*	HCC	↑	Downregulates miR-486/CDK4 expression; associated with poor OS	miR-486	Human; *in vivo*	-	+/+	Cheng et al., 2020
*circ_0078602*	HCC	↓	Associated with a poor prognosis	-	Human; *in vivo*	–	+/–	Kou et al., 2019
*circC3P1*	HCC	↓	Associated with TNM stage, tumor size and vascular invasion, overexpression decreased metastatic nodules	miR-4641	Human, Mouse; *in vitro*/*in vivo*	Human cell lines –BEL7402, Hep3B, HuH7, MHCC97-L, HL-7702	+/-	Zhong et al., 2018
*circ-ITCH*	HCC	↓	Correlated with poor OS	-	Human; *in vivo*	–	+/–	Guo et al., 2017
	BCa	↓	Inhibits cell proliferation, migration and invasion through circ-ITCH/miR-17,miR-224/p21,PTEN signaling axis	miR-17,miR-224	Human, Mouse; *in vitro*/*in vivo*	Human cell lines – EJ, T24, 253 J, RT4, TCC-SUP, UMUC, J82, 5637, SV-HUC	+/+	Yang C. et al., 2018
	CRC	↓	Overexpression reduces cell proliferation by downregulating c-Myc and cyclinD1	miR-7, miR-20a	Human; *in vitro*	Human cell lines – HCT116, SW480	+/+	Huang et al., 2015
*circMTO1*	HCC	↓	Promotes expression of a tumor suppressor p21 resulting in reduced tumor cell proliferation, metastasis and invasion	miR-9	Human, Mouse; *in vitro*/*in vivo*	Human cell lines – HepG2, SMMC-7721, QGY-7701, SK-Hep1	+/+	Han et al., 2017
*circ_0013520*	GBM	↑	Correlated with tumor size, TNM and worse OS	–	Human; *in vitro*	Human cell lines – SHG-44, U251, HEB	+/–	Zhou and Fan, 2020
*circ_0004379*	GBM	↑	Correlated with tumor size, TNM and worse OS	–	Human; *in vitro*	Human cell lines – SHG-44, U251, HEB	+/–	Zhou and Fan, 2020
*circ-CDC45*	GBM	↑	Associated with larger tumor size, higher grade, and worse survival	miR-516b, miR-527	Human; *in vitro*	Human cell lines – U87MG, U118, U251, LN229	+/-	Liu J. et al., 2019
*circNFIX*	GBM	↑	Predicts poor prognosis; promotes cell propagation and migration; knockdown enhances TMZ sensitivity in resistant cells; regulates NOTCH pathway	miR-132, miR-34a-5p	Human, Mouse; *in vitro*/*in vivo*	Human cell lines – HA1800, SF-539, SHG-44, U87	+/+	Xu et al., 2018; Ding et al., 2020
*circ_0013958*	LC	↑(LAC)	Associated with the TNM stage and lymphatic metastasis	miR-134	Human; *in vitro*	Human cell lines – A549, H1299, BEAS-2B	+/–	Zhu X. et al., 2017
*circFARSA*	LC	↑(NSCLC)	Promotes cell migration and invasion; upregulates FASN	miR-330-5p, miR-326	Human; *in vitro*/*in vivo*	Human cell line – A549	+/–	Hang et al., 2018
	CRC	↑	Promotes proliferation, migration, and invasion;regulates miR-330-5p/LASP1 axis	miR-330-5p	Human, Mouse; *in vitro*/*in vivo*	Human cell lines – FHC, LS174T, RKO, HT29, HCT116, SW480	+/+	Lu C. et al., 2020
*circ_0014130*	LC	↑(NSCLC)	Associated with tumor volume, distant metastasis;upregulates Bcl2	miR-136-5p	Human, Mouse; *in vitro*/*in vivo*	Human cell lines – PC-9, A549	+/–	Geng Y. et al., 2020
*circ_0000792*	LC	↑(LAD)	Overexpression is correlated with T stage, distant metastasis	–	Human; *in vivo*	–	+/–	Li, 2018
*circ_100876*	LC	↑(NSCLC)	Related to carcinogenesis of NSCLC and it might serve as a potential prognostic biomarker and therapeutic target	–	Human; *in vivo*	–	+/+	Yao J. T. et al., 2017
*circFADS2*	LC	↑	Associated with advanced TNM stage, lymph node metastasis, poor differentiation, and shorter OS; induces progression, invasion and proliferation	miR-498	Human; *in vitro*	Human cell line – HepG2	+/+	Zhao F. et al., 2018
	CRC	↑	Associated with distant metastasis	-	Human; *in vivo*	–	+/–	Xiao et al., 2020
*circPVT1*	LC	↑(NSCLC)	Associated with distant metastasis; promotes cell proliferation, migration and invasion, and inhibits apoptosis through upregulated E2F2 and E2F2-related protein expression	miR-125b	Human, Mouse; *in vitro*/*in vivo*	Human cell lines – A549, H292, SPC-A1, H1299, H1650, H1975, SK-MES-1, HBE	+/+	Li X. et al., 2018c
	OSC	↑	Associated to chemoresistance and lung metastasis; knockdown decreases ABCB1 expression, promotes chemoresistance; knockdown partly reverses the doxorubicin and cisplatin resistance	–	Human; *in vitro*	Human cell lines – SaoS2, KHOS, U2OS, MG63	+/+	Kun-peng et al., 2018b
	ALL	↑	Promotes cell proliferation and inhibits apoptosis	let-7,miR-125	Human; *in vivo, in vitro*	Human ALL cell lines	+/+	Hu et al., 2018
*circ_0067934*	LC	↑(NSCLC)	Tumor-promoting circRNA; induces cell proliferation, metastasis and invasion	–	Human; *in vitro*	Human cell lines – A549, H1299, SK-MES-1, PC-9, BEAS-2B	+/-	Wang and Li, 2018
	ESCC	↑	Promotes proliferation and migration	–	Human; *in vitro*	Human cell lines – TE-13, ECA-109	+/+	Zong et al., 2018b
	LSCC	↑	Promote cell proliferation and metastasis	miR-1324	Human; *in vitro*	Human cell lines – TU212, TU686, 16HBE	+/–	Chu, 2020
	HCC	↑	Enhances migration, invasion and proliferation of cells; regulates Wnt/β-catenin signaling pathway	miR-1324	Human; *in vitro*	Human cell lines – BEL7402, Hep3B, HuH7, MHCC97-L, HL-7702	+/+	Zhu et al., 2018
*circPRKCI*	LC	↑	Upregulation increased proliferation and tumorigenesis	miR-545, miR-589	Human, Mouse; *in vitro*/*in vivo*	Human cell lines – A549, NCI-H1975, NCI-H1703, NCI-H226, NCI-H46, PC9, NCI-H1299, SPC-A1, HCC827, HBE	+/–	Qiu et al., 2018
*circ_0000064*	LC	↑	Promotes cell proliferation and inhibits cell apoptosis, enhances expression of bcl-2; overexpression is correlated with TNM stage, lymph node metastasis,	–	Human; *in vitro*	Human cell lines – A549, H1229	+/+	Luo et al., 2017
*circ_0016760*	LC	↑(NSCLC)	miR-1287 directly targets GAGE1, higher expression associated with shorter OS, correlated with lymph node metastasis	miR-1287	Human, Mouse; *in vitro*/*in vivo*	Human cell lines – A549, H358, H1299, H1975	+/–	Li Y. et al., 2018
*circ_102231*	LC	↑(LAC)	Promotes lung cancer cells proliferation, migration and invasion	–	Cell line model (*in vitro* exp)	Human cell lines – BEAS-2B, A549	+/+	Zong et al., 2018a
*circRNA_103809*	LC	↑	Regulates miR-4302/ZNF121/MYC loop; promotes tumor growth, cell proliferation and invasion, associated with tumor stage and lymph node metastasis	miR-4302	Human, Mouse; *in vitro*/*in vivo*	Human cell lines – A549, H125, 95D, NCI-H292, H1975, HBE	+/+	Liu W. et al., 2018
	CRC	↓	Promote apoptosis through FOXO4 activity	miR-532-3p	Human; *in vitro*	Human cell lines – SW620, HCT116, COCA-2, HT29, FHC	+/+	Bian et al., 2018
*circ_0005962*	LC	↑(LAC)	–	–	Human; *in vivo*	–	+/–	Liu X. X. et al., 2019
*circ_0086414*	LC	↓(LAC)	Associated with EGFR mutations	–	Human; *in vivo*	–	+/–	Liu X. X. et al., 2019
*circ-PRMT5*	LC	↑(NSCLC)	Correlated with larger tumor, LNM, poor OS and progression free survival; upregulates EZH2	–	Human, Mouse; *in vitro*/*in vivo*	Human cell lines – HBE, A549, 95-D, HCC827, H1299, SK-MES-1	+/–	Wang Y. et al., 2019
	GC	↑	Promotes GC cell growth, clone formation, migration and invasion and inhibits apoptosis	miR-145, miR-1304	Human; *in vitro*	Human cell lines – AGS, MKN-28, MKN45, BGC823, MGC803, SGC7901, GES-1	+/+	Du et al., 2019
*circ-RAD23B*	LC	↑(NSCLC)	Regulates miR-593e3p/CCND2 axis; increases cell invasion *via* miR-653e5p/TIAM1 pathway	miR-593e3p, miR-653e5p	Human; *in vitro*	Human cell lines – 16HBE, H1299, H1581, H358, A549	+/+	Han et al., 2019
*circ_0102533*	LC	↑(NSCLC)	Associated with tumor type, TNM stages, lymph nodes metastasis and distant metastasis or recurrence	–	Human; *in vitro*	Human cell lines – A549, H1299, H1792, SK-MES-1, SPC-A1	+/–	Zhou X. et al., 2018
*circ_0079530*	LC	↑	Enhances cell proliferation and invasion	–	Human; *in vitro*	Human cell lines – A549, H1299, H460, Calu1, BEAS-2B	+/–	Li J. et al., 2018a
*circFGFR3*	LC	↑(NSCLC)	Increases cell invasion and proliferation, regulates Gal-1, pAKT, and p-ERK1/2	miR-22-3p	Human; *in vitro*	Human cell lines – 95C, 95D, A549, H460	+/–	Qiu B. Q. et al., 2019
*circ_000984*	LC	↑(NSCLC)	Promotes cell proliferation and metastasis; regulates Wnt/β-catenin signaling	–	Human; *in vitro*	Human cell lines – H1975, SPC-A1, H1299, HCC827, PC 9, A549, BEAS-2B	+/–	Li X. et al., 2019
*circ_0001946*	LC	↑(LAC)	Regulates SIRT1 that activates Wnt/β-catenin signaling pathway	miR-135a-5p	Human, Mouse; *in vitro*/*in vivo*	Human cell lines – H1299, A549, Calu3, SPC-A1, BEAS-2B	+/–	Yao et al., 2019
	GBM	↓	Reduces the migration, invasion, and proliferation of GBM cells	miR-671-5p	Human, Mouse; *in vitro*/*in vivo*	Human cell lines – U87, U251, HM	+/+	Li, 2019a
*circ_0037515*	LC	↓(NSCLC)	–	–	Human; *in vivo*	–	+/–	Zhao D. et al., 2020
*circ_0037516*	LC	↓(NSCLC)	–	–	Human; *in vivo*	–	+/-	Zhao D. et al., 2020
*circ_0033155*	LC	↓(NSCLC)	Reduces cell proliferation, colony formation and migration, correlated with lymphatic metastasis	–	Human; *in vitro*	Human cell lines – HCC827, H1975	+/+	Gu et al., 2018
*circ_100395*	LC	↓	Promotes LC malignancy regulating miR-1228/TCF21 axis	miR-1228	Human; *in vitro*	Human cell lines – A549, H460, Beas-2B	+/+	Chen D. et al., 2018
*circ-FOXO3*	LC	↓(NSCLC)	Promotes NSCLC development; releases FOXO3; miR-155 and FOXO transcription factors affect chemoresistance	miR-155	Human; *in vitro*	Human cell lines – A549, SPC-A1, NCI-H1299, NCI-H1650, SK-MES-1	+/+	Zhang Y. et al., 2018
*circ_0056616*	LC	↑(LAD)	Upregulation is correlated with TNM stage and lymph node metastasis	–	Human; *in vitro*	Human cell lines – PC9, PC14, HEK293T	+/–	He Y. et al., 2020
*circ_0010882*	GC	↑	Contributes to the proliferation of GC cells, migration, invasion, and apoptosis through modulating PI3K/Akt/mTOR pathway	–	Human; *in vitro*	Human cell lines – HGC-27, MKN-45, SGC-7901, BGC-823, GES-1	+/–	Peng et al., 2020
*circ-DCAF6*	GC	↑	Enhances GC progression	miR-1231, miR-1256	Human; *in vitro*	Human cell lines – AGS, BGC823, MGC803, GES1	+/–	Wu L. et al., 2019
*circ_0000419*	GC	↓	Associated with tumor stage, lymphatic and distal metastasis, venous and perineural invasion	hsa-miR-141-5p, hsa-miR-589-3p	Human; *in vitro*	Human cell lines – BGC-823, HGC-27, MGC-803, SGC-7901, GES-1	+/–	Tao et al., 2020
*circ_0006156*	GC	↓	Associated with lymph node metastasis, nerve invasion and degree of tumor differentiation	–	Human; *in vivo*	–	+/–	He F. et al., 2020
*circ_0001821*	GC	↓	Negatively associated with tumor depth and lymph node metastasis	–	Human; *in vitro*	Human cell lines – SGC-7901, HGC-27, BGC-823, AGS, MKN-1	+/-	Kong S. et al., 2019
*circCCDC9*	GC	↓	Upregulation sponges miR-6792-3p that targets CAV1, a tumor suppressor gene	miR-6792-3p	Human, Mouse; *in vitro*/*in vivo*	Human cell lines – GES-1, AGS, BGC-823, HGC-27, MGC-803, MKN-28, MKN-45, SGC-7901	+/+	Luo Z. et al., 2020
*circRHOBTB3*	GC	↓	Prevents the growth of cells, promotes expression of p21	miR-654-3p	Human, Mouse; *in vitro*/*in vivo*	Human cell lines – AGS, HGC27, MKN45	+/+	Deng et al., 2020
*circ_100269*	GC	↓	Resists GC development	miR-630	Human; *in vitro*	Human cell lines – AGS, MKN28, MKN45, BGC823, MGC803, SGC7901, GES1	+/+	Zhang Y. et al., 2017
*circ_0000745*	GC	↓	Associated with tumor differentiation	–	Human; *in vivo*	–	+/–	Huang M. et al., 2017
*circPSMC3*	GC	↓	Contributes to GC progression by regulating PTEN/miRNA-296-5p axis; PTEN regulates chemoresistance	miRNA-296-5p	Human, Mouse; *in vitro*/*in vivo*	Human cell lines – BGC823, MGC803, SGC7901, AGS, MKN45	+/-	Rong et al., 2019
*circ-KIAA1244*	GC	↓	Associated with TNM stage, lymphatic metastasis	–	Human; *in vivo*	–	+/–	Tang et al., 2018
*circYAP1*	GC	↓	Decreases GC cell growth and invasion; regulates miR-367-5p/p27 Kip1 axis	miR-367-5p	Human, Mouse; *in vitro*/*in vivo*	Human cell lines – GES-1, HGC-27	+/–	Liu H. et al., 2018
*circ_0006848*	GC	↓	Correlates with tumor differentiation and tumor size	–	–	–	+/–	Lu et al., 2019a
*circ_0000520*	GC	↓	Associated with TNM stage	–	Human; *in vitro*	Human cell lines – MKN-45, BGC-823, MGC- 80 803, AGS	+/–	Sun et al., 2017
*circ_0001895*	GC	↓	Associated with cell differentiation, Borrmann type, and tissue CEA expression	–	Human; *in vitro*	Human cell lines – GES-1, AGS, BGC-823, HGC27, MGC-803, SGC-7901	+/–	Shao et al., 2017
*circ_0005556*	GC	↓	Downregulation correlated with differentiation, TNM stage and lymphatic metastasis	–	Human; *in vivo*	–	+/–	Yang L. et al., 2019
*circ_0067582*	GC	↓	–	–	Human; *in vivo*	–	+/–	Yu et al., 2020
*circ_0000467*	GC	↑	Promotes proliferation, migration, and invasion of GC cells,inhibits tumor apoptosis	–	Human; *in vitro*	Human cell lines – HGC-27, MGC-803, AGS, NUGC-3, GES-1	+/–	Lu et al., 2019b
*circ_102958*	GC	↑	Overexpression is correlated with TNM stage	–	Human; *in vivo*	–	+/–	Wei et al., 2019
*circFUT8*	BCa	↓	Inhibits migration and invasion of Bca cells through silencing KLF10-mediated Slug signaling, inhibitory effect on lymphatic metastasis	miR-570-3p	Human, Mouse; *in vitro*/*in vivo*	Human cell lines – SV-HUC-1, T24, UM-UC-3	+/–	He Y. et al., 2020
*circ_0071662*	BCa	↓	Inhibits cell proliferation and invasion; upregulates HPGD and NF2	miR-146b-3p	Human; *in vivo*	–	+/+	Abulizi et al., 2019
*circ_0018289*	CC	↑	Associated with poor disease free survival	–	Human; *in vivo*	–	+/–	He Q. et al., 2020
*circ_0001038*	CC	↑	Promotes cell metastasis; suppresses inhibition of oncogenic targets like CNNM3 and MACC1	miR-337-3p	Human; *in vivo*	–	+/–	Wang Y. et al., 2020
*circEIF4G2*	CC	↑	Induce cell growth and migration	miR-218	Human; *in vitro*	Human cell lines – HeLa, CasKi, C33A, SiHa cells	+/–	Mao et al., 2019
*circCLK3*	CC	↑	Promotes cell proliferation, EMT, migration and invasion	miR-320a	Human, Mouse; *in vitro*/*in vivo*	Human cell lines – SiHa, HeLa, CaSki, C-33A, MS751	+/–	Hong et al., 2019
*circ_0000388*	CC	↑	Induces proliferation, migration, invasion,inhibit apoptosis; regulates miR-377-3p/TCF12 axis	miR-377-3p	Human; *in vitro*	Human cell lines – HeLa, SiHa	+/–	Meng et al., 2020
*circ_0101996*	CC	↑	–	–	Human; *in vivo*	–	+/–	Wang Y-M. et al., 2017
*circ_0101119*	CC	↑	–	–	Human; *in vivo*	–	+/–	Wang Y-M. et al., 2017
*circ_0104649*	CC	↑	–	–	Human; *in vivo*	–	–	Wang Y-M. et al., 2017
*circ_0104443*	CC	↑	–	–	Human; *in vivo*	–	–	Wang Y-M. et al., 2017
*circFoxO3a*	CC	↓	Correlates with stromal invasion, positive lymph node metastasis and poor prognosis	–	Human; *in vivo*	–	+/–	Tang et al., 2020
*circ_0081001*	OSC	↑	Overexpression was associated with poor prognosis	–	Human; *in vitro*	Human cell lines – MG63,KHOS,U2OS	+/–	Kun-peng et al., 2018a
*circ_0002052*	OSC	↓	Overexpression suppresses OS cell proliferation, migration and invasion while promoting apoptosis; regulates *circ_0002052*/miR-1205/APC2/Wnt/b-catenin pathway	miR-1205	Human; *in vitro*	Human cell lines – hFOB 1.19, 293T	+/+	Wu Z. et al., 2018
*circ-SLC7A5*	ESCC	↑	Overexpression correlated with TNM stage and poor OS	–	Human; *in vitro*	Human cell lines – K30, K70, K140, K180, K150, K450, T10, T12w	+/–	Wang Q. et al., 2020
*circ_0004771*	ESCC	↑	Increases the expression of CDC25	miR-339-5p	Human; *in vitro*	Human cell lines – FHC, HCT-116, SW480	+/–	Huang E. et al., 2020
	CRC	↑	–	–	Human; *in vitro*	Human cell lines – FHC, HCT-116, SW480	+/–	Pan et al., 2019
*circ_0092125*	OSCC	↓	Correlated with tumor size, TNM stage, and lymph node metastasis	-	Human; *in vitro*	Human cell lines – SCC15, SCC25, CAL27	+/–	Gao et al., 2020
*circ_0001874*	OSCC	↑	Associated with TNM stage and tumor grade	–	Human; *in vivo*	–	+/–	Zhao S. Y. et al., 2018
*circ_0001971*	OSCC	↑	Upregulation is associated with TNM stage	–	Human; *in vivo*	–	+/–	Zhao S. Y. et al., 2018
*circ-CCND1*	LSCC	↑	Improve the stability of CCND1 mRNA; increases LSCC growth	HuR; miR-646	Human, Mouse; *in vitro*/*in vivo*	Human cell lines –AMC-HN-8, Hep-2, LSC-1, TU212, TU177, TU686, SCC10A, NP-69	+/–	Zang et al., 2020
*circFLNA*	LSCC	↑	Induces migration of LSCC cells by targeting miR486-3p/FLNA axis; high level of FLNA implicates poor survival and drug resistance	miR486-3p	Human; *in vitro*	Human cell lines –Tu212, SCC-2, SCC40	+/+	Wang J. X. et al., 2019
*circMATR3*	*HSCC*	↑	Upregulation of oncogene USP28 that contributes to MYC stability	miR-188-5p, miR-448	Human; *in vitro*	Human cell line - FaDu	+/–	Wang Z. et al., 2020
*circMORC3*	*HSCC*	↓	Associated with T stages and tumor sizes	–	–	–	+/–	Zheng and Chen, 2020
*circMYBL2*	AML	↑	Increases the translational efficiency of FLT3 kinase; knockdown impairs the cytoactivity of FLT3-ITD AML cells, including quizartinib-resistant cells	–	Human, Mouse; *in vitro*/*in vivo*	Human cell lines – MV4-11, MOLM-13,THP-1, HL60, NB4 and ML-2,U937	+/+	Sun et al., 2019
*Circ-DLEU2*	AML	↑	Induces cell proliferation and reduces apoptosis	miR-496	Human, Mouse; *in vitro*/*in vivo*	Human cell lines – MOLM-13, HL-60, MV-4-11	+/+	Wu D. M. et al., 2018
*f-circPR*	AML	↑	Promoted cell proliferation	–	–	–	+/+	Guarnerio et al., 2016
*f-circM9*	AML	↑	Favors leukemia progression	–	–	–	+/+	Guarnerio et al., 2016
*circ_001569*	CRC	↑	Promotes cell proliferation and invasion	miR-145	Human; *in vitro*	Human cell lines – SW480, HCT116, SW620, LOVO	+/+	Xie et al., 2016
*circ_0007534*	CRC	↑	Increase in the Bcl2/Bax ratio in CRC cells and inhibits apoptosis	–	Human; *in vitro*	Human cell lines – SW620, HCT116, LoVo, SW480, HT29	+/+	Zhang R. et al., 2018
*circ_0000069*	CRC	↑	Knockdown induces G0/G1 arrest and inhibits cancer progression	–	Human; *in vitro*	Human cell lines – HT29, LoVo, HCT-116, SW480	+/+	Guo et al., 2016
*circ_0020397*	CRC	↑	Upregulates TERT and thereby induces cell proliferation	miR-138	Human; *in vitro*	Human cell lines – LoVo, HCT116, SW480, SW620	+/+	Zhang X. et al., 2017
*circBANP*	CRC	↑	Promotes CRC cell proliferation; induces p-Akt protein expression	–	Human; *in vitro*	Human cell lines – HT29, HCT116	+/+	Zhu M. et al., 2017
*circ5615*	CRC	↑	promotes CRC progression through miR-149-5p/TNKS axis	miR-149-5p	Human, Mouse; *in vitro*/*in vivo*	Human cell lines – HCT 116, LoVo, HT-29, SW480, NCM460	+/+	Ma et al., 2020
*circPTK2*	CRC	↑	Promotes EMT of CRC cells *via* expression of mesenchymal marker vimentin	–	Human, Mouse; *in vitro*/*in vivo*	Human cell lines – HCT15, SW620, SW480, LOVO	+/+	Yang H. et al., 2020
	LC	↓(NSCLC)	Overexpression augments T1F1γ expression, reduces TGF β induced EMT	miR-429/miR-200b3p	Human, Mouse; *in vitro*/*in vivo*	Human cell lines – BEAS-2B, A549, H1299, H1650, SPC-A1, Calu3,H226, H520, SK-MES-1	+/+	Wang L. et al., 2018
*circ_0060745*	CRC	↑	Promotes CSE1L-mediated CRC cell proliferation and metastasis	miR-4736	Human; *in vitro*	Human cell lines – NCM460, HT29, LOVO, PKO, SW480	+/+	Wang and Wang, 2020
*circ_0008285*	CRC	↓	Inhibits CRC cell proliferation and migration; regulates PI3K/AKT pathway	miR-382-5p	Human; *in vitro*	Human cell lines – SW480, RKO, HCT8, SW620, HCT116, DLD1,FHC	+/+	Wang et al., 2020
*circ-0001313*	CRC	↑	Inhibits apoptosis regulating PI3K/Akt signaling pathway	miRNA-510-5p	Human; *in vitro*	Human cell lines – SW620, HCT116, SW480, HT-29, LoVo, NCM460	+/+	Tu et al., 2020
*circDDX17*	CRC	↓	inhibits cell proliferation, migration, invasion, and promotes apoptosis	hsa-miR-21-5p	Human; *in vitro*	Human cell lines – SW480, SW620, HT29, LoVo, HCT116, RKO	+/+	Li X-N et al., 2018
*circ-ABCB10*	BC	↑	Knockdown suppresses proliferation and induces apoptosis	miR-1271	Human; *in vitro*	Human cell lines – MCF-7, MDA- MB-231, MDA-MB-468, MDA-MB-453	+/+	Liang et al., 2017
*circEHMT1*	BC	↓	Inhibits metastasis by regulating circEHMT1/miR-1233-3p/KLF4 axis	miR-1233-3p	Human, Mouse; *in vitro*/*in vivo*	Human cell lines – ZR-75-1, MCF-7, MB-468, T47D, SK-BR3, MDA-MB-231, BT-549, HMEpC	+/+	Lu M. et al., 2020
*circ_0011946*	BC	↑	Promotes migration and invasion	miR26a/b	Human; *in vitro*	Human cell lines – HS-578T, T47D, MCF-7, BT549, MDA-MB-231, SKBR-3	+/+	Zhou J. et al., 2018
*circGFRA1*	BC	↑(TNBC)	Promotes cell proliferation and inhibits apoptosis; regulates GFRA1 expression	miR-34a	Human, Mouse; *in vitro*/*in vivo*	Human cell lines – MCF10A, SKBR3, T47D, BT474, MCF-7, BT-483, BT-20, BT549, MDA-MB-468, MDA-MB-231	+/+	He et al., 2017
*circ_0001982*	BC	↑	Promotes tumorigenesis	miR-143	Human; *in vitro*	Human cell lines – MDA-MB-231, MCF-7, MDAMB-468, MDA-MB-435s	+/+	Tang et al., 2017
*circTADA2A*	BC	↓	Possesses tumor-suppressor capability, restores the expression of SOCS3, suppressed cell proliferation, migration, invasion, clonogenicity	miR-203a-3p	Human, Mouse; *in vitro*/*in vivo*	Human cell lines – MCF-7 MDA-MB-231	+/+	Xu et al., 2019
*circ-10720*	HCC	↑	Promotes migration, invasion and EMT by stabilizing vimentin	miR-1246, miR-578, miR-490-5p	Human, Mouse; *in vitro*/*in vivo*	Human cell lines – PLC-PRF-5, SMMC-7721, HEK-293T	+/+	Meng et al., 2018
*circPTGR1*	HCC	↑	Knockdown promotes expression of epithelial markers and reduces the levels of mesenchymal markers	miR-449a	Human, Mouse; *in vitro*/*in vivo*	Human cell lines – HepG2, L-O2, SMCC7721, HEP3B, HUH7, MHCC97-L, MHCC 97H, HCC-LM3	+/+	Wang G. et al., 2019
*circTRIM33–12*	HCC	↓	Upregulates TET1 expression; suppresses tumor proliferation, migration, invasion	miR-191	Human, Mouse; *in vitro*/*in vivo*	Human cell lines – MHCC97-L, HCC97-H, HCCLM3, SMMC-7721	+/+	Zhang P. F. et al., 2019
*circ-BIRC6*	HCC	↑	Knockdown reduces Bcl2 mRNA and protein levels	miR3918	Human, Mouse; *in vitro*/*in vivo*	Human cell lines – SKHEP-1, Huh-7 MHCC97H	+/+	Tang et al., 2015
*circ_0070269*	HCC	↓	Increases expression of NPTX1,that inhibits aggressive tumor behavior	miR182	Human, Mouse; *in vitro*/*in vivo*	Human cell lines – Hep3B, SMMC-7721, HepG2, PLC, Huh-7,LO2	+/+	Zhang P. F. et al., 2019
*circADAMTS13*	HCC	↓	Acts as a tumor suppressant; inhibits HCC proliferation	miR-484	Human; *in vitro*	Human cell lines – PLC/PRF/5, SK-Hep-1, Hep3B, HepG2	+/+	Qiu L. et al., 2019
*cZNF292*	GBM	↑	Promotes angiogenesis; regulates STAT3/5/β-catenin pathway	–	Human; *in vitro*	Human cell lines – U87MG and U251	+/+	Yang P. et al., 2019
*circ_0037251*	GBM	↑	Enhances GBM progression, upregulates mTOR; inhibits cell apoptosis and G1 phase arrest	miR-1229-3p	Human, Mouse; *in vitro*/*in vivo*	Human cell lines – U373, U251, HEK293T	+/+	Cao et al., 2019
*circMAPK4*	GBM	↑	Suppress apoptosis through decreased phosphorylation of p38/MAPK	miR-125a-3p	Human, Mouse; *in vitro*/*in vivo*	Human cell lines – U138, U373, U87	+/+	He et al., 2020a
*circ-U2AF1*	GBM	↑	Enhances cell proliferation, migration, and invasion, increases expression of NOVA2	miR-7-5p	Human, Mouse; *in vitro*/*in vivo*	Human cell lines – U87MG, U251, U87, HEB	+/+	Li, 2019a
*circNT5E*	GBM	↑	Upregulates NT5E, SOX4, PI3KCA; promotes cell proliferation, migration, and invasion	miR-422a	Human, Mouse; *in vitro*/*in vivo*	Human cell lines – U87, U251	+/+	Wang R. et al., 2018a
*circ_0029426*	GBM	↑	Promotes cell proliferation, migration and invasion, and inhibits cell apoptosis	miR-197	Human; *in vitro*	Human cell lines – U87, U251, LN229, U87MG, A172, NHA	+/+	Zhang G. et al., 2019
*circ-TTBK2*	GBM	↑	Regulates *circ-TTBK2*/miR-217/HNF1β/Derlin-1 axis; promotes cell proliferation, migration, and invasion, while inhibiting apoptosis	miR-217	Human, Mouse; *in vitro*/*in vivo*	Human cell lines – U87, U251, HEK293T	+/+	Zheng et al., 2017
*circMMP9*	GBM	↑	Upregulates the expression of (CDK4) and aurora kinase A; promotes proliferation, migration and invasion abilities	miR-124	Human, Mouse; *in vitro*/*in vivo*	–	+/+	Wang R. et al., 2018b
*circ_0020123*	LC	↑(NSCLC)	Upregulates ZEB1 and EZH2 for tumor growth and EMT	miR-144	Human, Mouse; *in vitro*/*in vivo*	Human cell lines – PC9, H1573, A549, SK-MES-1, H1299, Calu-3	+/+	Qu et al., 2018
*f-circEA-4a*	LC	↑(NSCLC)	Induces cell proliferation, metastasis and invasion	–	Human; *in vitro*	Human cell lines –A549, HT1299	+/+	Tan et al., 2018
*f-circEA-2a*	LC	↑(NSCLC)	Promotes cell migration and invasion	–	Human; *in vitro*	Human cell lines –A549, HT1299	+/+	Tan et al., 2018
*circ_104916*	GC	↓	Inhibits cell proliferation, migration and EMT	–	Human, Mouse; *in vitro*/*in vivo*	Human cell lines – AGS, MKN-28, NCI-N87, MKN-45,GES1	+/+	Li J. et al., 2017
*circPDSS1*	GC	↑	Enhances expression of NEK2 leading to cell migration and proliferation	miR-186-5p	Human; *in vitro*	Human cell lines – MGC-803, HGC-27, BGC-823, GES-1	+/+	Ouyang et al., 2019
*circ_0023642*	GC	↑	Promotes cell proliferation and metastasis; upregulates N-cadherin, Vimentin and Snail expression	–	Human; *in vitro*	Human cell lines – MGC-803, MNK-45,SGC-7901, HGC-27, GES1	+/+	Zhou L. H. et al., 2018
*circATAD1*	GC	↑	Increases cell progression, upregulates YY1	miR-140-3p	Human; *in vivo, in vitro*	Human cell lines – GES1, SGC7901, BGC-823, AGS, MGS-803	+/+	Zhang L. et al., 2020
*circFN1*	GC	↑	Promotes viability and inhibits apoptosis; facilitates CDDP resistance *in vitro*	miR-182-5p	Human, Mouse; *in vitro*/*in vivo*	Human cell lines – SGC7901CDDP, BGC823C DDP, SGC7901, SGC823)	+/+	Huang X. X. et al., 2020
*circCACTIN*	GC	↑	Induces EMT and regulates Smad signaling, promote metastatic conversion, angiogenesis	miR-331-3p	Human, Mouse; *in vitro*/*in vivo*	Human cell lines – GES1, BGC-823, MGC-803, SGC-7901c	+/+	Zhang L. et al., 2019
*circ-CEP85L*	GC	↓	Inhibits tumor growth, proliferation and invasion of GC cells	miR-942-5p	Human, Mouse; *in vitro*/*in vivo*	Human cell lines – MGC-803, AGS, KATOIII, BGC-823, HGC-27, MKN-45	+/+	Lu J. et al., 2020
*circMYLK*	BCa	↑	Augments proliferation, migration, the tube formation of HUVEC, and EMT; stabilizes VEGFA	miR-29a	Human, Mouse; *in vitro*/*in vivo*	Human cell lines – EJ, T24, 5673, BIU-87	+/+	Zhong et al., 2017
*circACVR2A*	BCa	↓	Upregulates EYA4 expression; suppresses proliferation, migration and invasion and metastasis through miR-626/EYA4 axis	miR-626	Human, Mouse; *in vitro*/*in vivo*	Human cell lines – T24, UM-UC-3, RT4, J82, 5637, HT-1376, TCCSUP, SV-HUC-1	+/+	Dong et al., 2019
*circ_0061140*	Ovarian cancer	↑	Regulates miR370/FOXM1 pathway; promotes cell proliferation, migration, and the EMT	miR-370	Human, Mouse; *in vitro*/*in vivo*	Human cell lines – SKOV3, A2780, OV2008, IGROV1, ES-2	+/+	Chen Q. et al., 2018
*circUBAP2*	OSC	↑	Stable expression of Bcl-2; promotes OS growth and inhibits apoptosis	miR-143	Human, Mouse; *in vitro*/*in vivo*	Human cell lines – hFOB 1.19, MG63, U2OS	+/+	Zhang H. et al., 2017
*circ_001564*	OSC	↑	Inhibits cell cycle arrest in G0/G1 phase and apoptosis	miR-29c-3p	Human; *in vitro*	Human cell lines – U2OS, Saos-2, HOS, MG-63	+/+	Song and Li, 2018
*circNASP*	OSC	↑	Augments FOXF1 expression that leads to proliferation and invasion of OS cells, positively correlates with the tumor size and metastasis	miR-1253	Human; *in vitro*	Human cell lines – 143B and MG63	+/+	Huang et al., 2018
*circ_0000337*	ESCC	↑	Promotes cell proliferation, migration, and invasion	miR-670-5p	Human; *in vitro*	Human cell lines – KYSE-150, TE-1, HET-1A	+/+	Song et al., 2019
*circUHRF1*	OSCC	↑	Modulates the transcription factor c-Myc; regulates circUHRF1/miR-526b-5p/c-Myc/TGF-β1/ESRP1 axis	miR-526b-5p	Human, Mouse; *in vitro*/*in vivo*	Human cell lines – SCC25, CAL27, SCC15, TSCCA	+/+	Zhao W. et al., 2020
*circ_0082182*	CRC	↑	Correlated with lymph node metastasis	–	Human; *in vitro*	Human cell lines – HCT116, SW480, SW620, NCM460	+/–	Ye D. et al., 2019
*circ_0000370*	CRC	↑	Correlated with lymph node metastasis	–	Human; *in vitro*	Human cell lines – HCT116, SW480, SW620, NCM460	+/–	Ye D. et al., 2019
*circ_0035445*	CRC	↓	Correlated with TNM stage	–	Human; *in vitro*	Human cell lines – HCT116, SW480, SW620 and normal cell line-NCM460	+/–	Ye D. et al., 2019
*circ5615*	CRC	↑	Promotes CRC progression through miR-149-5p/TNKS axis	miR-149-5p	Human, Mouse; *in vitro*/*in vivo*	Human cell lines – HCT 116, LoVo, HT-29, and SW480, NCM460 and HEK-293T	+/+	Ma et al., 2020
*circ_0060745*	CRC	↑	Regulates miR-4736/SCE1L	miR-4736	Human; *in vitro*	Human cell lines – human colon epithelial cell line (NCM460) and human CRC cell lines (HT29, LoVo, PKO, and SW480)	+/–	Wang and Wang, 2020
*circ_0026344*	CRC	↓	Correlated with metastasis	miR-21,miR-31	Human, Mouse; *in vitro*/*in vivo*	Human cell lines – HCT116, SW480, HT29, SW620, NCM460, FHC, HEK293T	+/–	Yuan et al., 2018
*circ_0000567*	CRC	↓	Correlated with lymph metastasis, distal metastasis and TNM stage	–	Human; *in vitro*	Human cell lines – FHC, SW480, RKO, CACO2, SW620, HCT116	+/–	Wang J. et al., 2018
*circ_0003906*	CRC	↓	Correlated with poor differentiation and lymphatic metastasis	–	Human; *in vitro*	Human cell lines: NCM460, SW480, SW620, HCT8, HCT116, HT29, LoVo	+/–	Zhuo et al., 2017

*AML, Acute Myeloid Leukemia; CML, Chronic Myeloid Leukemia; CLL, Chronic Lymphocytic Leukemia; MM, Multiple Myeloma; BCL, B cell Lymphoma; CRC, Colorectal Cancer; BC, Breast Cancer; HCC, Hepatocellular Carcinoma; GBM, Glioblastoma; LC, Lung Cancer; NSCLC, Non-Small Cell Lung carcinoma; LAC, Lung Adenocarcinoma; GC, Gastric Cancer; BCa, Bladder Cancer; CC, Cervical Cancer; OSC, Osteosarcoma; ESCC, Esophageal Squamous Cell Cancer; OSCC, Oral Squamous Cell Carcinoma; LSCC, Laryngeal Squamous Cell Cancer; HSCC, Hypopharyngeal Squamous Cell Carcinoma*.

### Hematological Malignancies

#### Acute Myeloid Leukemia (AML)

*Circ_0009910* is overexpressed in the bone marrow of AML patients, which correlates with poor overall survival (OS) (Ping et al., 2019a). It sponges miR-20a-5p and knocking it down induces apoptosis in AML cells (Ping et al., 2019a). *Circ-vimentin* is upregulated in AML patients and its elevated expression is an independent poor prognostic factor for OS and leukemia-free survival (LFS) in AML patients (Yi and Lin, 2018). Hirsh et al. examined the expression of *circ_0075001* in a cohort of NPM1 wild-type and mutated AML patients and found it to be positively correlated with expression of the cognate linear RNA but independent of the NPM1 mutational status (Hirsch et al., 2017). However, high *circ_0075001* expression levels defined patient subgroups characterized by lower expression of components of the Toll-like receptor (TLR) signaling pathway which is associated with a more immature AML phenotype (Hirsch et al., 2017). C*irc_0004277* was downregulated in AML patients expression, however, its expression is restored in AML patients subjected to chemotherapy indicating it as a potential diagnostic marker and treatment target in AML (Li W. et al., 2017).

#### Chronic Myeloid Leukemia (CML)

C*irc_100053* was upregulated in peripheral blood mononuclear cells and serum of CML patients and was associated with clinical stage and BCR/ABL mutation status (Ping et al., 2019b). Elevated *CircRNA_100053* levels predicted a poor outcome in CML patients and imatinib resistance (Ping et al., 2019b).

#### Chronic Lymphocytic Leukemia (CLL)

*Circ-RPL15* was upregulated in CLL patients and correlated with poor OS and immunoglobulin heavy-chain variable region (IGHV) mutation used in the validation of CLL prognosis (Wu Z. et al., 2019). *Circ-RPL15* sequesters miR-146b-3p and activates RAS/RAF1/MEK/ERK pathway to promote CLL development (Wu Z. et al., 2019). *Circ-CBFB* levels are also elevated in CLL patients and its expression can distinguish CLL patients from healthy controls (Xia L. et al., 2018). It sponges miR-607, which targets FZD3, an activator of Wnt/β-catenin signaling in CLL (Xia L. et al., 2018). Higher expression of *circ-CBFB* predicted reduced OS in CLL patients and may serve as a prognostic marker for CLL (Xia L. et al., 2018).

#### Multiple Myeloma (MM)

Elevated expression of *circ_0007841* in MM correlated with chromosomal aberrations such as gain 1q21, t (4:14), mutations in ATR, and IRF4 genes, however, its function in MM needs further investigation (Gao et al., 2019). Feng et al. observed lower levels of *circ_0000190* in MM tissues and peripheral blood, which correlated with the prognosis and OS of MM patients (Feng et al., 2019). C*irc-SMARCA5* is downregulated in MM and its higher expression correlates with lower β2-microglobulin (MG) level and less advanced International Staging System stage (Liu H. et al., 2019). C*irc-SMARCA5* downregulation correlates with reduced OS, progression-free survival (PFS), and treatment response in MM patients (Liu H. et al., 2019). *CircRNA_101237* level was upregulated in MM patients and has high diagnostic accuracy for MM (Liu and Wang, 2020). Its expression was elevated in patients with 13q14 deletion, 1q21 amplification, p53 deletion, and t(4,14) and t(14,16) gene mutations, but was decreased in those with t(11,14) gene mutations; also the upregulation was associated with a poor response to chemotherapy (Liu and Wang, 2020).

#### B Cell Lymphoma (BCL)

*Circ-APC (circ_0127621)* was downregulated in diffuse large B-cell lymphoma (DLBCL) and its levels in plasma can distinguish patients from healthy controls (Hu et al., 2019). Moreover, DLBCL patients with lower *circ-APC* levels were more likely to exhibit an advanced Ann Arbor stage, shorter OS, resist chemotherapy and display a low International Prognostic Index (Hu et al., 2019).

### Solid Tumors

#### Colorectal Cancer (CRC)

*CDR1as* was upregulated in CRC tissues and its overexpression correlated with poor survival (Weng et al., 2017). Its upregulation is an independent risk factor for OS and enhanced EGFR/RAF1/MAPK pathway by inhibiting miR-7 tumor-suppressor activity (Weng et al., 2017). Similarly, *circCCDC66* was also elevated in CRC patients and its high expression correlated with poor prognosis (Hsiao et al., 2017). It sponges miRNA-33b and miR-93 to promote CRC growth and metastasis by stabilizing MYC mRNA (Hsiao et al., 2017). Increased expression of *circ_0004585* in the CRC tissues was associated with increased tumor size (Tian et al., 2019). *Circ_0007142* (Zhu et al., 2019). *Circ_0007142* regulates invasion of CRC by sponging miR-103a-2-5p and its upregulation, associated with poor differentiation and lymphatic metastasis (Zhu et al., 2019). Upregulation of *circ-HUEW1* in CRC tissues was associated with lymphovascular invasion, lymph node and distant metastasis, and Tumor, Node, and Metastasis (TNM) stage (Chen H. Y. et al., 2020). It sponges miR-486 and regulates the IGF2/β-catenin signaling pathway by targeting PLAGL2 (Chen H. Y. et al., 2020). CRC patients with higher *circ_0001178* were more likely to have metastatic features, advanced TNM stage, and adverse prognosis (Ren et al., 2020). It sponges miR-382, miR-587, and miR-616, all of which target ZEB1 (Ren et al., 2020). *Circ_0005075* was also highly expressed in CRC and is associated with depth of invasion and advanced TNM stage, and is a prognostic factor affecting both OS and disease-free survival (DFS) in CRC patients (Jin et al., 2019). *CircHIPK3* is upregulated in CRC tissues and serves as an independent prognostic factor of poor OS and positively correlates with metastasis and advanced clinical stage (Zeng et al., 2018). *Circhipk3* sponges miR-7 and promotes CRC progression by increasing the expression of its target genes FAK, IGF1R, EGFR, and YY1 (Zeng et al., 2018). Ye et al. discovered a 3-circRNA signature as a non-invasive biomarker for CRC diagnosis, they observed elevated expression of *circ_0082182* and *circ_0000370*, and downregulated *circ_0035445* levels in the plasma of CRC patients (Ye D. et al., 2019). The upregulation of *circ_0082182* and *circ_0000370* was strongly associated with lymph node metastasis, while the *circ_0035445* downregulation was connected with the TNM stage (Ye D. et al., 2019). Also, *circ_0082182* and *circ_0035445* showed a difference between preoperative and postoperative stages, while *circ_0000370* had no significant difference between these two stages (Ye D. et al., 2019). *Circ_0007534* was upregulated in plasma of CRC patients, which correlated with the progression of clinical classifications, metastatic phenotype, poor differentiation, and poor prognosis in CRC patients (Zhang W. et al., 2018). Increased *circ_0007534* expression was associated with poor prognosis in CRC patients (Zhang W. et al., 2018). Overexpression of *circFADS2* was closely related to the size, differentiation, infiltration depth, lymphatic and distant metastasis, and TNM stage of CRC patients (Xiao et al., 2020). Patients with increased *circFADS2* levels had a poor OS and had a better predictive value when combined with the TNM stage (Xiao et al., 2020). *Circ5615* was upregulated in CRC was an independent prognosis factor for CRC, and it was associated with a higher T stage and poor OS in CRC patients (Ma et al., 2020). Patients with elevated expression of *circ-FARSA* had a poor OS and the circRNA promotes CRC progression by regulating the miR-330-5p/LASP1 axis (Lu C. et al., 2020). Overexpression of *circ_0060745* in CRC tissues was significantly associated with shorter OS, advanced clinical stage, nodal classification, metastasis classification, and liver metastasis (Wang and Wang, 2020). It promoted CRC progression by sponging miR-4736 and regulating SCE1L expression (Wang and Wang, 2020). Downregulation of *circ_0001649* in CRC tissues and patient serum was negatively associated with CRC differentiation (Ji et al., 2018). *CircITGA7* was downregulated in CRC tissues and was negatively associated with tumor size, lymph metastasis, distant metastasis, and TNM stage (Li X. et al., 2018a). It sponges miR-370-3p and inactivates Ras signaling pathway by upregulating neurofibromin 1 (NF1) (Li X. et al., 2018a). *Circ_0000711* was downregulated in CRC tissues and could act as a diagnostic marker for CRC (Li J. et al., 2018b). Downregulation of *circ_0014717* in CRC tissues was associated with distant metastasis, TNM stage, and poor OS (Wang F. et al., 2018). Its overexpression promoted cell-cycle arrest by increasing p16 expression leading to reduced growth and invasion of CRC cells (Wang F. et al., 2018). *Circ_0004771* was upregulated in serum exosomes of CRC patients and had good diagnostic potential (Pan et al., 2019). *Circ_0026344* was downregulated in CRC samples of stage III/IV as compared to tissues with stage I/II (Yuan et al., 2018). Lower *circ_0026344* expression correlated with metastasis and predicted poor prognosis in CRC patients (Yuan et al., 2018). *Circ_0000567* expression was downregulated in CRC tissues and was associated with tumor size, lymph metastasis, distal metastasis, and TNM stage (Wang J. et al., 2018). *Circ_0003906* was downregulated in CRC tumors and its lower expression was associated with a higher incidence of poor differentiation, lymphatic metastasis, and is an independent risk factor for survival of CRC patients (Zhuo et al., 2017). Analysis of serum from CRC patients revealed overexpression of *exo-circ-PNN* and it can serve as a potential non-invasive biomarker for CRC detection (Xie Y. et al., 2020).

#### Breast Cancer (BC)

C*ircFBXW7* was downregulated in triple-negative breast cancer (TNBC) and was correlated with poor clinical outcomes (Ye F. et al., 2019). Its expression was also negatively associated with tumor size, lymph node metastasis, and is an independent prognostic factor for TNBC (Ye F. et al., 2019). *CircFBXW7* sponges miR-197-3p and also encodes for a microprotein FBXW7-185aa that upregulates the tumor-suppressor, FBXW7 (Ye F. et al., 2019). The upregulation of *circSEPT9* in TNBC tissues was associated with advanced clinical stage and poor prognosis (Zheng et al., 2020). It decoys miR-637 and modulates, leukemia inhibitory factor (LIF) expression to activate TNBC progression (Zheng et al., 2020). *Circ_0001785* was upregulated in BC plasma samples and correlated with histological grade, TNM stage, and distant metastasis (Yin et al., 2018). Autophagy-associated *circCDYL* was upregulated in tissues and serum from BC patients (Liang et al., 2020). Higher *circCDYL* levels were associated with estrogen receptor (ER) negative status, higher Ki67 index, larger tumor size, and more lymphatic metastasis (Liang et al., 2020). Moreover, BC patients with high serum *circCDYL* had a poorer OS compared to early BC and benign patients (Liang et al., 2020). *CircKIF4A* was overexpressed in TNBC tissues, which correlated with tumor size, lymph node metastasis, and TNM stage (Tang et al., 2019). It induced TNBC cell proliferation and migration by sponging miR-375 and regulating KIF4A expression (Tang et al., 2019). The higher expression levels of *circKIF4A* correlated with poor OS in TNBC patients (Tang et al., 2019). *CircPLK1* was also upregulated in TNBC tissues and correlated with larger tumor size, lymph node positivity, advanced TNM stage and poor OS (Kong Y. et al., 2019). It promotes TNBC metastasis by sponging miR-296-5p and regulating PLK1 expression (Kong Y. et al., 2019). Song et al. observed a higher expression of cytoplasmic *circHMCU* in BC tissues (Song et al., 2020). *CircHMCU* sequestered members of let-7 family and enhanced proliferation and metastasis in BC (Song et al., 2020). Its upregulation was associated with histological grade, lymph node metastasis, TNM stage, and poor prognosis (Song et al., 2020). Downregulation of *circ_0068033* in BC tissues was associated with tumor size and TNM stage (Yuan et al., 2020). It sequesters miR-659 and its overexpression induces apoptosis (Yuan et al., 2020). Overexpression of *circGFRA1* in TNBC tissues was associated with tumor size, TNM staging, lymph node metastasis, and histological grade (He et al., 2017). Patients with upregulated *circGFRA1* had shorter OS and DFS (He et al., 2017). *CircGFRA1* increases proliferation and inhibit apoptosis by regulating the expression of its cognate gene GFRA1 by sponging miR-34a (He et al., 2017). *CircTADA2A-E6* and *circTADA2A-E5/E6* generated from the *TADA2A* gene were downregulated in BC (Xu et al., 2019). Lower expression of *circTADA2A-E6* was associated with increased lymphatic metastasis and advanced clinical stage (Xu et al., 2019). BC patients with downregulated *circTADA2A-E6* had a poor prognosis with shorter DFS and OS, whereas no association was identified between DFS or OS and *circTADA2A-E5/E6* levels (Xu et al., 2019).

#### Hepatocellular Carcinoma (HCC)

The upregulation of *circ_0005075* in HCC tissues was associated with tumor size and had good diagnostic potential (Shang et al., 2016). It decoys miR-23b-5p, miR-93-3p, miR581, and miR-23a-5p and contributes to HCC proliferation, invasion, and metastasis (Shang et al., 2016). Overexpression of *circ_100338* was related to low OS and metastatic progression in HCC patients with HBV infection (Huang X. Y. et al., 2017). It decoys miR-141-3p and increases metastatic progression in HCC (Huang X. Y. et al., 2017). *CircRHOT1* was upregulated in HCC, its expression was higher in stage III HCC tissues than in stage I/II (Wang L. et al., 2019). HCC patients with higher *circRHOT1* expression had poor prognosis (Wang L. et al., 2019). *Circ_0091579* was overexpressed in HCC tissues and its upregulation was associated with poor OS of HCC patients (Zhang C. et al., 2018). Interestingly, exposure of HCC samples to cisplatin upregulated *circRNA_101237*, and its expression correlated with tumor size, lymph node metastasis, distant metastasis, and TNM stage (Zhang C. et al., 2018). *CircRNA_101237* was upregulated in HCC tissues and serum samples, and was correlated with tumor size, lymph node metastasis, distant metastasis, and TNM stage (Zhou et al., 2020). Elevated serum *circRNA_101237* levels was an independent predictor of poor OS and prognosis in HCC patients (Zhou et al., 2020). *Circ-HOMER1* was also upregulated in HCC tissues and associated with larger tumor size, higher TNM stage, and poor prognosis (Zhao M. et al., 2020). It decoys miR-1322 and upregulates CXCL6 (Zhao M. et al., 2020). *Circ_0016788* was upregulated in HCC tissues and was associated with poor OS, higher performance status score, larger tumor size, increased Barcelona clinic liver cancer (BCLC) stage, abnormal aspartate aminotransferase, abnormal alpha-fetoprotein and abnormal carbohydrate antigen 199 levels (Cheng et al., 2020). *CDR1as* was upregulated in HCC samples and was one of the independent factors of hepatic microvascular invasion and had the potential predictive ability (Xu et al., 2017). *CDR1as* promotes HCC progression by activating PI3K/AKT/mTOR pathway by sponging miR-7 (Xu et al., 2017). *Circ_0078602* was downregulated in HCC tissues and was associated with poor prognosis (Kou et al., 2019). *CircC3P1* was also downregulated in HCC and was negatively correlated with TNM stage, tumor size, vascular invasion, and lower OS in HCC patients (Zhong et al., 2018). It sponges miR-4641 which targets PCK1 (Zhong et al., 2018). *Circ_0001649* was downregulated in HCC and the decreased expression associated with tumor size and occurrence of tumor embolus (Qin et al., 2016). Guo et al. observed the downregulation of *circ-ITCH* in HCC tissues, which correlated with the poor OS, whereas upregulated *circ-ITCH* associated with favorable survival in HCC patients (Guo et al., 2017). *CircMTO1* was downregulated in HCC tissues and its decreased expression was associated with poor prognosis of HCC patients (Han et al., 2017). Downregulation of *circTRIM33-12* was observed in HCC tissues and associated with tumor proliferation, migration, invasion, and immune evasion, and it also served as an independent risk factor for OS and recurrence-free survival (RFS) of HCC patients after surgery (Zhang P. F. et al., 2019). *CircTRIM33-12* reduces HCC metastasis and immune evasion by upregulating TET1 expression by sponging miR-191 (Zhang P. F. et al., 2019). *CircADAMTS13* was downregulated in HCC tissues and this correlated with the absence of liver cirrhosis, larger tumor size, more severe BCLC stage, and poor patient prognosis (Qiu L. et al., 2019). *CircADAMTS13* serves as a tumor-suppressor by sponging miR-484 (Qiu L. et al., 2019). *Circ_0070269* was downregulated in HCC tissues and its low expression was correlated with advanced TNM stage, large tumor size, lymph node metastasis, poor OS, and metastasis-free survival of HCC patients (Xiaotong et al., 2019). *Circ_0070269* inhibits HCC progression by regulating the miR-182/NPTX1 axis (Xiaotong et al., 2019). Lower expression levels of *circSMARCA5* in tissues and plasma samples of HCC patients has good diagnostic potential (Li Z. et al., 2019). Downregulation of *circSMARCA5* was associated with tumor differentiation, TNM stage, cancer invasion, and cancer diameter (Li Z. et al., 2019). Adipose-secreted exo-circ-deubiquitination (*circ-DB*) was upregulated in HCC patients with higher body fat ratios (Zhang H. et al., 2019). It promotes HCC growth and reduces DNA damage by suppression of miR-34a and the activation of USP7 (Zhang H. et al., 2019). Depletion of *circ-DB* suppressed HCC growth and metastasis *in vivo* (Zhang H. et al., 2019).

#### Glioblastoma (GBM)

Lyu et al. using circRNA microarrays identified several differentially expressed circRNAs in GBM (Zhou and Fan, 2020). Enhanced expression of *circ_0013520* and *circ_0004379* correlated with tumor size, TNM stage, and worse OS in GBM patients (Zhou and Fan, 2020). *Circ-CDC45* was also elevated in GBM and associated with larger tumor size, higher grade, and poor OS in glioma (Liu J. et al., 2019). *Circ-CDC45* serves as a sponge for miR-516b and miR-527 which functions as tumor-suppressor in GBM (Liu J. et al., 2019). Exosomal *circNFIX* was upregulated in the serum of temozolomide (TMZ) resistant patients and predicted poor prognosis (Ding et al., 2020). It sequesters miR-132 in GBM cells and its knockdown enhanced TMZ-sensitivity (Ding et al., 2020). *Circ_0029426* was upregulated in GBM tissues and this was associated with tumor size and World Health Organization grading (Zhang G. et al., 2019). C*irc_0029426* was an independent prognostic factor for GBM and correlated with the poor OS (Zhang G. et al., 2019). It promotes GBM progression by sequestering miR-197 (Zhang G. et al., 2019).

#### Lung Cancer (LC)

*Circ_0013958* was upregulated in lung adenocarcinoma (LUAD) tissues and plasma of patients and was associated with TNM stage and lymphatic metastasis (Zhu X. et al., 2017). *Circ_0013958* decoys miR-134 and upregulates CCND1 in LUAD (Zhu X. et al., 2017). *CircFARSA* was upregulated in tissues and plasma of non-small-cell lung carcinoma (NSCLC) patients (Hang et al., 2018). High expression of *circFARSA* correlated with cell migration and invasion (Hang et al., 2018). *CircFARSA* sequesters miR-330-5p and miR-326, leading to the upregulation of the oncogene fatty acid synthase (FASN) (Hang et al., 2018). *Circ_0014130* was also overexpressed in NSCLC tissues and correlated with tumor volume, distant metastasis, and poor prognosis (Geng Y. et al., 2020). Li et al. observed the upregulation of *circ_0000792* in LUAD tissues, which correlated with T stage, distant metastasis, and smoking status (Li, 2018). Overexpression of *circ_100876* in NSCLC tissues was correlated with tumor stage, lymph node metastasis, and reduced OS in NSCLC patients (Yao J. T. et al., 2017). *Circ_100876* acts by sequestering miR-136 which targets MMP13 (Yao J. T. et al., 2017). Microarray analysis revealed the upregulation of *circFADS2* in LC tissues and this correlated with advanced TNM stage, lymph node metastasis, poor differentiation, and shorter OS of NSCLC patients (Zhao F. et al., 2018). *CircFADS2* induces NSCLC progression by sponging miR-498 (Zhao F. et al., 2018). Overexpression of *circPVT1* in NSCLC tissues and serum samples was associated with distant metastasis (Li X. et al., 2018c). *Circ_0067934* was upregulated in NSCLC tissues and its overexpression was correlated with TNM stage, lymph node status, and distant metastasis (Wang and Li, 2018). Overexpression of *circ_0067934* is associated with poorer OS and is an independent poor prognostic factor for NSCLC patients (Wang and Li, 2018). *CircPRKCI* was upregulated in LUAD tissues and associated with tumor size, TNM stage, poor prognosis, and shorter OS (Qiu et al., 2018). Higher *circPRKCI* increased proliferation and tumorigenesis of LUAD by sponging miR-545 and miR-589 and upregulating E2F7 (Qiu et al., 2018). *Circ_0000064* was upregulated in LUAD tissues and its higher expression levels correlated with T stage, lymphatic metastasis and TNM stage (Luo et al., 2017). Increased *circ_0000064* inhibited Caspase-3, Caspase-9, and Bax, and enhanced Bcl-2 expression in LUAD (Luo et al., 2017). Overexpression of *circ_0016760* in LUAD tissues correlated with TNM stage, lymph node metastasis, smoking status, differentiation grade and shorter OS; promotes NSCLC development by sponging miR-1287 that targets G-antigen 1 (GAGE1) and is an independent predictor for the survival of NSCLC patients after surgery (Li J. et al., 2018a). Yao et al. reported the upregulation of *circRNA_100876* in NSCLC tissues and it was associated with lymph node metastasis, tumor staging, and shorter OS (Yao J. T. et al., 2017). *CircRNA_102231* overexpression in LUAD tissues correlated with advanced TNM stage (III-IV), lymph node metastasis, and poor OS (Zong et al., 2018a). C*ircRNA_103809* was significantly overexpressed in LUAD tissues and its higher expression correlated with a poor OS (Liu W. et al., 2018). *CircRNA_103809* enhanced LUAD progression by regulating the miR-4302/ZNF121/MYC loop (Liu W. et al., 2018). *Circ_0005962* was appreciably upregulated and *circ_0086414* was downregulated in early-stage LUAD and this 2-circRNA signature is a promising diagnostic biomarker for early LUAD (Liu X. X. et al., 2019). Higher plasma levels of *circ_0086414* were associated with EGFR mutations (Liu X. X. et al., 2019). Upregulation of *circ-PRMT5* were observed in NSCLC tissues and they were associated with larger tumors, lymph node metastasis, later clinical TNM stage, poor OS and PFS in NSCLC patients and is an independent prognostic factor for NSCLC patients (Wang Y. et al., 2019). *Circ-RAD23B* overexpression in NSCLC tissues was associated with lymph node metastasis, lower differentiation grade, and poor OS (Han et al., 2019). *Circ-RAD23B* enhanced cell growth by regulating the miR-593-3p/CCND2 axis and increased cell invasion by regulating the miR-653e5p/TIAM1 pathway (Han et al., 2019). *Circ_0102533* was elevated in NSCLC tissues and whole blood samples up and its regulation was significantly associated with tumor type, TNM stages, lymph nodes metastasis, and distant metastasis or recurrence (Zhou X. et al., 2018). *Circ_0102533* was useful in the detection of stage I-II NSCLC patients and elevated *circ_0102533* levels in whole blood was acceptable as a blood-based tumor marker for NSCLC screening (Zhou X. et al., 2018). *Circ_0079530* functions as an oncogene in NSCLC by enhancing cell proliferation and invasion and its overexpression was associated with tumor size and lymph node metastasis (Li J. et al., 2018a). *CircFGFR3* was significantly upregulated in LC tissues and its overexpression was closely associated with poor prognosis and reduced OS after surgery (Qiu B. Q. et al., 2019). *CircFGFR3* increased NSCLC cell invasion and proliferation by regulating Gal-1, pAKT, and p-ERK1/2 by sponging miR-22-3p (Qiu B. Q. et al., 2019). Elevated *circ_000984* levels in NSCLC tissues correlated with advanced TNM stage, lymph nodes metastasis, poor OS, and lower DFS in NSCLC patients (Li X. et al., 2019). *Circ_000984* activated Wnt/β-catenin signaling and its overexpression is an independent prognostic indicator for NSCLC patients (Li X. et al., 2019). Overexpression of *circ_0001946* in LUAD tissues was associated with a higher TNM stage, tumor size, and low OS (Yao et al., 2019). *Circ_0001946* enhanced LUAD progression by sponging miR-135a-5p and stabilizing its target SIRT1, which activates Wnt/β-catenin signaling pathway (Yao et al., 2019). *Circ_0037515* and *circ_0037516* were significantly downregulated in NSCLC tissues and have the potential for diagnosis (Zhao D. et al., 2020). Reduced levels of *circ_0033155* in NSCLC tissue was associated with lymphatic metastasis, and its overexpression reduced cell proliferation, colony formation and migration, and increased the level of PTEN in NSCLC (Gu et al., 2018). Downregulation of *circ_100395* in LC tissues was associated with metastasis and poor prognosis (Chen D. et al., 2018). Overexpression of *circ_100395* reduced malignancy by regulating the miR-1228/TCF21 axis (Chen D. et al., 2018). Downregulation of *circ_0001649* in NSCLC tissues was associated with positive lymph node, smoking status, and differentiation grade (Liu T. et al., 2018). Patients with downregulated *circ_0001649* had shorter OS and it could be a prognostic biomarker for NSCLC (Liu T. et al., 2018). *CircRNA_0056616* was upregulated in tissues and plasma of LUAD patients and it was correlated with TNM stage and lymph node metastasis (He F. et al., 2020). *Circ_0000190* and *circ_000164* were overexpressed in plasma and tissues from LC patients and expression of *circ_0000190* was associated with late-stage, extra-thoracic metastasis, poor survival, and prognosis (Luo Y. H. et al., 2020). These exosomal circRNAs are easily detectable in liquid biopsy and may serve as potential biomarkers for LC (Luo Y. H. et al., 2020).

#### Gastric Cancer (GC)

Deregulation of circRNAs has been reported in many gastric cancers and they can potentially serve as useful prognostic markers and therapeutic targets (Naeli et al., 2020). Elevated *CDR1as* levels in GC tissues was an independent risk factor and linked to the poor OS in GC patients (Pan et al., 2018). *CDR1as* enhances the development of GC by activating PTEN/PI3K/AKT pathway by sponging miR-7 (Pan et al., 2018). Overexpression of *circ_0010882* in the plasma of GC patients was a prognostic factor for OS and correlated with the poor OS (Peng et al., 2020). *Circ_0010882* contributes to GC cells proliferation, migration, invasion, and apoptosis by modulating PI3K/AKT/mTOR pathway (Peng et al., 2020). The upregulation of *circ-DCAF6* was associated with depth of invasion, lymph node invasion, and TNM stage in GC patients and is an independent risk factor for OS (Wu L. et al., 2019). *Circ-PRMT5* was upregulated in GC tissues and it was associated with tumor size, TNM stages, degree of differentiation, lymph node metastasis, and distant metastasis (Wu L. et al., 2019). GC patients with reduced *circPRMT5* expression had better prognosis and OS than those with increased levels (Wu L. et al., 2019). *Circ-PRMT5* promoted GC cell growth, migration, and invasion by sponging miR-145 and miR-1304 and upregulating MYC expression (Du et al., 2019). *Circ_0009910* expression was significantly increased in GC tissues and correlated with clinical stage, distant metastasis, and differentiation (Liu M. et al., 2018). Patients with elevated *circ_0009910* had a poor OS compared to patients with decreased expression (Liu M. et al., 2018). *Circ_0000419* was downregulated in GC plasma and exosomes and this negatively correlated with tumor stage, lymphatic and distal metastasis, venous, and perineural invasion (Tao et al., 2020). *Circ_0000419* is predicted to sponge miR-141-5p and miR-589-3p and its downregulation significantly correlate with Borrmann type and differentiation grade (Tao et al., 2020). Patients with downregulated *circ_0000419* had a poor OS and DFS (Tao et al., 2020). Downregulation of *circ_0006156* in GC tissues was associated with lymph node metastasis, nerve invasion, and degree of tumor differentiation, besides low expression of *circ_0006156* correlated with progression-free survival, and OS of GC patients (He Y. et al., 2020). *Circ_0001821* was significantly downregulated in GC tissues, and whole-blood specimens of GC patients (Kong S. et al., 2019). Downregulation of *circ_0001821* was negatively associated with tumor depth and lymph node metastasis (Kong S. et al., 2019). The combined use of circulating *circ_0001821* with the existing tumor markers yielded good diagnostic potential in GC (Kong S. et al., 2019). Downregulation of *circCCDC9* in GC tissues was negatively associated with tumor size, lymph node invasion, advanced clinical stage, and OS (Luo Z. et al., 2020). *CircCCDC9* sponges miR-6792-3p which targets CAV1 a tumor-suppressor gene in GC (Luo Z. et al., 2020). Downregulation of *circRHOBTB3* in GC tissues was associated with poor differentiation and unfavorable prognosis in GC patients (Deng et al., 2020). *CircRHOBTB3* has a tumor-suppressor activity and inhibits growth of GC cells by sponging miR-654-3p and promoting the expression of its target p21 (Deng et al., 2020). *CircRNA_100269* was downregulated in GC tissues and its lower expression was associated with histological subtypes and node invasion (Zhang Y. et al., 2017). GC patients with low *circRNA_100269* levels had poor OS than patients with higher levels (Zhang Y. et al., 2017). Downregulation of *circRNA_100269* promoted GC development by releasing its inhibitory effect on oncogenic miR-630 (Zhang Y. et al., 2017). *Circ_0000745* was downregulated in GC tissues and plasma samples of GC patients and was associated with tumor differentiation and TNM stage (Huang M. et al., 2017). The use of *circ_0000745* in plasma combined with carcinoembryogenic antigen showed potential for use as a diagnostic marker for GC (Huang M. et al., 2017). Downregulation of *circPSMC3* was observed in plasma and tissue samples from GC patients and is negatively correlated with TNM stage, lymphatic metastasis, and reduced OS in GC patients (Rong et al., 2019). *CircPSMC3* contributed to GC progression by regulating PTEN by sponging miRNA-296-5p (Rong et al., 2019). *Circ-KIAA1244* was downregulated in plasma and tissues from GC patients and was negatively associated with the TNM stage, lymphatic metastasis, and reduced OS (Tang et al., 2018). Downregulation of *circ-KIAA1244* was an independent prognostic indicator of OS for GC patients (Tang et al., 2018). Downregulation of *circ_0000190* was observed in tissues and plasma samples of GC patients and is correlated with tumor diameter, lymphatic metastasis, distal metastasis, TNM stage, and CA19-9 levels (Chen et al., 2017). Chen et al. observed downregulation of *circSMARCA5* in GC tissues and it correlated with differentiation, lymph node metastasis, vascular invasion, poor OS and DFS in GC patients, moreover, low *circSMARCA5* expression was an independent prognostic factor for survival of GC patients (Cai et al., 2019). *CircYAP1* was downregulated in GC tissues and was correlated with poor prognosis and reduced OS in GC patients (Liu H. et al., 2018). *CircYAP1* expression was higher in early-stage GC patients and such patients were more sensitive to chemotherapy (Liu H. et al., 2018). *CircYAP1* decreased cell growth and invasion by sponging miR-367-5p to upregulate p27 ^Kip1^ (Liu H. et al., 2018). Lower expression of *circ_0006848* in GC tissues correlated with tumor differentiation and tumor size (Lu et al., 2019a). Levels of *circ_0000520* were also decreased in tissues and plasma of GC patients and correlated negatively with the TNM stage in tissues and with CEA expression in plasma (Sun et al., 2017). *Circ_0001895* was significantly downregulated in GC tissues and its lower expression was associated with cell differentiation, Borrmann type, and CEA expression (Shao et al., 2017). *Circ_0005556* was downregulated in GC tissues and its low expression closely correlated with poor differentiation, TNM stage, and lymphatic metastasis (Yang L. et al., 2019). GC patients with decreased *circ_0005556* levels had a shorter OS than those with higher levels (Yang L. et al., 2019). *Circ_0067582* was downregulated in GC tissues and is correlated with increased tumor diameter and high CA19-9 (Yu et al., 2020). *Circ_0067582* downregulation was associated with a better prognosis after surgery (Yu et al., 2020). *Circ_0000467* was overexpressed in GC tissue and plasma and this was correlated with the TNM stage (Lu et al., 2019b). Diagnostic potential of *circ_0000467* was found to be superior to other common plasma biomarkers such as CEA and carbohydrate antigens-724 (CA-724) (Lu et al., 2019b). Elevated *circRNA_102958* levels were observed in GC tissues and it was significantly correlated with the TNM stage (Wei et al., 2019). Overexpression of *circ-ATAD1* was observed in GC and associated with deeper invasion, positive lymph node metastasis, advanced TNM stages, and adverse prognosis (Zhang L. et al., 2020). It promotes GC tumorigenesis by regulating the miR-140-3p/YY1 signaling axis (Zhang L. et al., 2020). *CircSHKBP1* was overexpressed in tumors and serum exosomes of GC patients, and it correlated with advanced pathological staging and poor OS (Xie M. et al., 2020). *CircSHKBP1* promotes GC progression by sponging miR-582-3p to increase HuR levels and promoting VEGF stability, and also by binding HSP90 to prevent its interaction with STUB1 (Xie M. et al., 2020).

#### Bladder Cancer (BCa)

Downregulation of *circFUT8* in BCa tissues was correlated with poor prognosis, high histological grade, lymph node metastasis, and poor survival rate (He Q. et al., 2020). C*irc_0071662* was downregulated in BCa tissues and this correlated with poor prognosis, lymph node invasion and distal metastasis, and poor OS (Abulizi et al., 2019). Overexpression of *circ_0071662* inhibited cell proliferation and invasion by sponging miR-146b-3p and upregulating its targets, hydroxy prostaglandin dehydrogenase (HPGD) and neurofibromin 2 (NF2) (Abulizi et al., 2019). *Circ-ITCH* was downregulated in BCa and this was associated with the histological grade of BCa patients (Yang C. et al., 2018). BCa patients with decreased *circ-ITCH* expression had poor OS than those with higher levels (Yang C. et al., 2018). Upregulation of *circ-ITCH* inhibited cell proliferation, migration, and invasion through *circ-ITCH*/miR-17, miR-224/p21, PTEN signaling axis (Yang C. et al., 2018). *CircACVR2A* was downregulated in BCa tissues and cell lines and its downregulation was correlated with advanced pathological stage, high grade, lymphatic metastasis, and poor OS (Dong et al., 2019). C*ircACVR2A* reduces proliferation, migration, and invasion of BCa cells by sponging miR-626 to regulate EYA4 expression (Dong et al., 2019).

#### Cervical Cancer (CC)

*Circ_0018289* was upregulated in CC tissues and this correlated with tumor size and lymph node metastasis and poor DFS in CC patients (He et al., 2020b). Overexpression of *circ_0001038* in CC tissues was associated with lymph node invasion, myometrial invasion, and unfavorable outcome (Wang Y. et al., 2020). It promotes metastasis by sequestering miR-337-3p and upregulating it targets, Cyclin A, CBS Domain Divalent Metal Cation Transport Mediator 3 (CNNM3), and Metastasis Associated In Colon Cancer 1 (MACC1) (Wang Y. et al., 2020). *CircEIF4G2* was upregulated in CC tissues and this correlated with tumor size and lymph node metastasis (Mao et al., 2019). Elevated expression of *circEIF4G2* was correlated with worse prognosis in CC patients and induced cell growth and migration by sponging miR-218 and increasing the expression of its target HOXA1 (Mao et al., 2019). Increased expression of *circCLK3* in CC tissues was associated with poor tumor differentiation, advanced International Federation of Gynecology and Obstetrics (FIGO) stages and depth of stromal invasion, and indicated poor OS and DFS (Hong et al., 2019). It decoys miR-320a to remove its suppressive effects on FoxM1 and promotes cell proliferation, EMT, migration, and invasion (Hong et al., 2019). Higher *circ_0000388* levels in CC patients were significantly associated with FIGO stage, lymph node metastasis, and depth of invasion (Meng et al., 2020). *Circ_0000388* increased the proliferation, migration, and invasion, and reduced apoptosis of CC through regulating the miR-377-3p/ TCF12 axis (Meng et al., 2020). Wang et al. observed that 4 circRNAs namely, *circ_0101996, circ_0104649, circ_0104443*, and *circ_0101119* were significantly upregulated in peripheral whole blood from CC patients (Wang Y-M. et al., 2017). Combined detection of *circ_0101996* and *circ_0101119* could easily distinguish CC patients from healthy controls (Wang Y-M. et al., 2017). *CircFoxO3a* was significantly downregulated in the serum of CC patients and correlated with deep stromal invasion, positive lymph node metastasis, and poor prognosis (Tang et al., 2020). C*ircFoxO3a* downregulation is a poor prognostic factor for both OS and recurrence-free survival, independent of positive lymph node metastasis in CC patients (Tang et al., 2020).

#### Osteosarcoma (OSC)

C*irc_0081001* was overexpressed in OSC tissues and serums samples and was associated with poor prognosis, and may serve as an independent prognostic factor and biomarker for OSC diagnosis and prognosis (Kun-peng et al., 2018a). *Circ_0002052* was also upregulated in OSC tissues and associated with advanced stage, tumor size, metastasis, and poor survival rate in OSC patients (Jing et al., 2020). *Circ-0002052* promotes OSC development by activating Wnt/β-catenin signaling by sponging miR-382 (Jing et al., 2020). *CircPVT1* was significantly upregulated in OSC tissues and serum samples (Kun-peng et al., 2018b). Moreover, levels of *circPVT1* were higher in patients with lung metastasis or chemoresistance (Kun-peng et al., 2018b). Increased expression of *circPVT1* correlated with advance Enneking stage, chemoresistance, and lung metastasis, and was found to be a better diagnostic marker than alkaline phosphatase (ALP) for OSC (Kun-peng et al., 2018a). *CircHIPK3* was downregulated in OSC tissues and plasma samples (Xiao-Long et al., 2018). Lower *circHIPK3* levels correlated with Enneking stage, lung metastasis, lower OS, and poor prognosis in OSC patients (Xiao-Long et al., 2018). *Circ_0000190* was found in the extracellular nanovesicles and transmitted from healthy cells to OSC cells to impede cancer development (Li et al., 2020). Reduced expression of *circ_0000190* correlated with bigger tumor size, advanced staging (IIB/III), and distant metastasis and is a potential biomarker for OSC (Li et al., 2020).

### Head and Neck Squamous Cell Cancer (HNSCC)

#### Esophageal Squamous Cell Cancer (ESCC)

Overexpression of *circ-SLC7A5* in ESCC plasma samples was correlated with TNM stage and poor OS (Wang Q. et al., 2020). Elevated *circ-0004771* levels were associated with heavier tumor burden and poor prognosis (Huang E. et al., 2020). *Circ_0067934* was upregulated in ESCC tissues and its increased expression correlated with poor differentiation, I-II T stage, and I-II TNM stage (Zong et al., 2018b).

#### Oral Squamous Cell Carcinoma (OSCC)

Lower expression of *circ_0092125* in OSCC correlated with tumor size, TNM stage, and lymph node metastasis in OSCC patients (Gao et al., 2020). Downregulation of *circ_0092125* was associated with shorter OS and was an independent risk factor for OSCC prognosis (Gao et al., 2020). Zhao et al. compared circRNAs levels in the saliva between OSCC patients and healthy donors, and observed upregulation of *circ_0001874* and *circ_0001971* in the saliva of OSCC patients and this correlated with tumor stage and TNM (Zhao S. Y. et al., 2018).

#### Laryngeal Squamous Cell Carcinoma (LSCC)

*Circ_0067934* was upregulated in LSCC tissues and its overexpression was associated with larger tumor size, stronger lymph node metastasis, distant metastasis, and poor prognosis with lower OS rate (Chu, 2020). Upregulated *circ-CCND1* levels in LSCC correlated with tumor size, poor differentiation, advanced TNM stage, and poor prognosis (Zang et al., 2020). It binds to HuR and miR-646 to enhance the stability of CCND1 mRNA (Zang et al., 2020). *CircFLNA* upregulation in LSCC was associated with lymph node metastasis (Wang J. X. et al., 2019). *CircFLNA* increased the migration of LSCC cells by targeting the miR486-3p/FLNA axis (Wang J. X. et al., 2019).

#### Hypopharyngeal Squamous Cell Carcinoma (HSCC)

*CircMATR3* was upregulated in HSCC tissues and was associated with advanced clinical stage, poor lymph node metastasis, and poor survival of HSCC patients (Wang Z. et al., 2020). *CircMATR3* binds to miR-188-5p and miR-448, both having a common target, USP28 (Wang Z. et al., 2020). *CircMORC3* downregulation in HSCC tissues and plasma samples was associated with tumor stage and tumor size (Zheng and Chen, 2020).

## Circular RNAs in Cancer Therapeutics

Recent advances in RNA-based therapeutics coupled with aberrant expression of circRNAs in cancers makes them attractive therapeutic tools (Liu et al., 2017; Yang Z. et al., 2017; Lei et al., 2019). For example, circRNAs with multiple binding sites for oncogenic proteins or miRNAs can be introduced exogenously to restore the normal regulatory network to control proliferation and apoptosis in cancer (Tay et al., 2015). To facilitate this, multiple strategies to manipulate circRNA levels are currently under investigation and have good prospect for being developed into circRNA-based therapeutic strategies in near future.

The easiest approach to inhibit circRNA expression is RNA interference, using small interfering RNAs (siRNAs), short hairpin RNAs (shRNAs) or by employing chemically modified antisense oligonucleotides (ASOs) complementary to the back-splice junction, latter is preferred for *in vivo* applications (Cortés-López and Miura, 2016; Santer et al., 2019). Furthermore, complete knockdown of circRNA, *CDR1as*, by CRISPR/Cas9 genome-editing has been achieved and *CDR1as* loss-of-function mutant mice were generated (Piwecka et al., 2017). Another possibility is use of the CRISPR/Cas13 RNA knockdown system, wherein circRNA silencing is attained by targeting the CRISPR/Cas13 guide RNA to the back-splice junction of the circRNA (Santer et al., 2019). CircRNA overexpression is usually achieved by retroviral, lentiviral, adenoviral, or adeno-associated virus (AAV) vector constructs bearing circRNA sequence flanked by introns containing intronic complementary sequences (ICS) and splicing signals (Wang K. et al., 2017; Bai et al., 2018; Xia P. et al., 2018). Additionally, antisense oligonucleotides (ASOs) can also be used to enhance circRNA expression, by targeting splice-sites or splice-enhancers to increase the efficiency of back-splicing (Zhang et al., 2014). Apart from this, non-viral systems for circRNA overexpression have also been explored, most notable being *in vitro* synthesis of circRNAs followed by their *in vivo* delivery. Exogenous circRNA production first involves the synthesis of linear RNA by *in vitro* transcription, followed by circularization by employing self-splicing introns or by T4 RNA ligase (Santer et al., 2019). CircRNAs with therapeutic potential are discussed below and summarized in Table 1.

### Hematological Malignancies

#### Acute Myeloid Leukemia (AML)

*CircMYBL2* is significantly upregulated in AML patients with FLT3-ITD mutations and it increases the translational efficiency of FLT3 transcript, by facilitating binding of polypyrimidine tract binding protein 1 (PTBP1) to FLT3 transcript (Sun et al., 2019). Downregulation of *circMYBL2* reduced levels of FLT3 kinase and inhibited proliferation and promoted differentiation of FLT3-ITD AML (Sun et al., 2019). Overexpression of *circ-DLEU2* promoted AML by sponging miR-496 and increasing levels of its target, PRKACB (Wu D. M. et al., 2018). Tumor growth due to overexpression of *circ-DLEU2 in vivo* was reversed by its knockdown (Wu D. M. et al., 2018). Guarnerio et al. demonstrated that the well-established oncogenic chromosomal translocations such as *PML/RAR*α and *MLL/AF9* give rise to fusion circRNAs (f-circRNA), *f-circPR*, and *f-circM9*, respectively (Guarnerio et al., 2016). Expression of these f-circRNAs in mouse embryonic fibroblasts promoted cell proliferation and transformed foci-forming capability (Guarnerio et al., 2016). Consistent with its role in promoting cell proliferation, knockdown of *f-circM9* increased apoptosis in AML cells (Guarnerio et al., 2016). Presence of *f-circM9* conferred protection to leukemic cells in a mice model upon treatment by arsenic trioxide and cytarabine (Guarnerio et al., 2016).

#### Acute Lymphoid Leukemia (ALL)

Higher expression of *circ-PVT1* contributes to ALL progression by sponging let-7 and miR-125 (Hu et al., 2018). Knockdown of *circ-PVT1* inhibits cell proliferation and induces apoptosis by reducing expression of c-Myc and Bcl-2, which are targets of let-7 and miR-125 respectively (Hu et al., 2018).

#### Chronic Myeloid Leukemia (CML)

*Circ_0009910* was upregulated in CML and promotes imatinib resistance by sequestering miR-34a-5p which targets ULK1 (Cao et al., 2020). Knockdown of *circ_0009910* reduced cell growth and imatinib resistance, along with increased apoptosis and autophagic activation (Cao et al., 2020).

#### Multiple Myeloma (MM)

*Circ-CDYL* facilitated MM growth by sponging miR-1180 and increasing the expression of its target YAP (Chen F. et al., 2020). Downregulation of *circ-CDYL* induces apoptosis by downregulating YAP (Chen F. et al., 2020).

#### B-Cell Lymphoma (BCL)

*Circ-APC* was significantly downregulated in DLBCL (Hu et al., 2019). In cytoplasm *circ-APC* sponges miR-888 leading to an increase in levels of its target APC, whereas in nucleus it binds to APC promoter and recruits the DNA demethylase TET1 to transcriptionally upregulate APC (Hu et al., 2019). Ectopically expressed *circ-APC* acts as a tumor-suppressor and acts by inhibiting Wnt/β-catenin signaling in DLBCL (Hu et al., 2019).

### Solid Tumors

#### Colorectal Cancer (CRC)

*CDR1as* which acts as miR-7 RNA sponge is overexpressed in CRC and confers an aggressive oncogenic phenotype (Weng et al., 2017). *CDR1as* downregulation resulted in inhibition of CRC progression (Weng et al., 2017). EGFR expression is regulated by *circHIPK3* which is upregulated in CRC tissues (Zeng et al., 2018). Similar to *CDR1as, circHIPK3* also functions as a sponge for miR-7. The knockdown of *circHIPK3* inhibited cell proliferation, migration, invasion, and metastasis (Zeng et al., 2018). *Circ_001569* was significantly upregulated in CRC tissues and promoted cell proliferation and invasion (Xie et al., 2016). Mechanistically, *circ_001569* performs a tumor-promoting function by sponging miR-145 and upregulating its targets E2F5, BAG4, and FMNL2 (Xie et al., 2016). Downregulation of *circ*_*001569* resulted in reduced cell invasion and migration (Xie et al., 2016). *Circ_0007534* upregulation was associated with a metastatic phenotype and evasion of apoptosis in CRC (Zhang R. et al., 2018). Silencing of *circ_0007534* reduced Bcl2/Bax ratio in CRC cells and induced apoptosis (Zhang R. et al., 2018). Levels of *circ_0000069* were also elevated in CRC tissues and its knockdown induced cell-cycle arrest and inhibited cancer progression (Guo et al., 2016). *Circ_ITCH* acts as a sponge for miR-7 and miR-20a and is significantly downregulated in CRC tissues (Huang et al., 2015). Overexpression of *circ_ITCH* reduced cell proliferation in CRC by downregulating c-Myc and cyclinD1 (Huang et al., 2015). *CircRNA_103809* is also downregulated in CRC patients and its silencing promotes cell proliferation and migration *via* miR-532-3p/FOXO4 axis (Bian et al., 2018). Interestingly, telomerase reverse transcriptase (TERT) is one of the targets of the tumor-suppressor miR-138 which is sponged by *circ_0020397*(Zhang X. et al., 2017). *Circ_0020397* is upregulated in CRC tissues and its downregulation resulted in lower TERT levels and reduced cell proliferation (Zhang X. et al., 2017). *CircBANP* was significantly upregulated in CRC tissues and cell lines and its silencing suppressed CRC cell proliferation and reduced p-Akt protein expression (Zhu M. et al., 2017). *Circ5615* is upregulated in CRC tissues and functions by sequestering miR-149-5p which targets tankyrase (TNKS), an activator of Wnt/β-catenin stabilization (Ma et al., 2020). Downregulation of *circ5615* inhibited proliferation and promoted cell-cycle arrest (Ma et al., 2020). *CircFARSA* is upregulated in CRC tissues and sequesters miR-330-5p, leading to the upregulation of LASP1 (LIM and SH3 protein 1) (Lu C. et al., 2020). The silencing of *circFARSA* inhibited proliferation, migration, and invasion of CRC cells (Lu C. et al., 2020). *CircPTK2* is elevated in CRC tissues and functions by promoting EMT of CRC cells by binding to vimentin protein at Serine 38, 55, and 82 residues (Yang H. et al., 2020). *CircPTK2* knockdown reduced tumorigenicity and metastatic potential of CRC cells (Yang H. et al., 2020). *Circ_0060745* promotes CRC metastasis by sequestering miR-4736 and stabilizing its target CSE1L (chromosome segregation 1-like) (Wang and Wang, 2020). The knockdown of *circ_0060745* suppressed CRC cell migration and invasion (Wang and Wang, 2020). *Circ_0008285* is downregulated in CRC tissues and cell lines (Wang and Wang, 2020). It acts by suppressing PI3K/AKT signaling *via* miR-382-5p/PTEN axis, leading to inhibition of cell proliferation and migration in CRC (Wang and Wang, 2020). *Circ_0001313* is highly expressed in CRC tissues and modulates tumorigenesis by sponging miR-510-5p to elevate AKT2 expression (Tu et al., 2020). Depletion of *circ-0001313* decreased proliferation and induced apoptosis in CRC cells (Tu et al., 2020). *CircDDX17* is significantly downregulated in CRC tissues and its silencing promoted CRC cell proliferation, migration, invasion, and inhibited apoptosis (Li X-N et al., 2018).

#### Breast Cancer (BC)

*Circ-ABCB10* sponges miR-1271 in BC, its depletion suppresses proliferation and induces apoptosis in BC cells (Liang et al., 2017). *CircEHMT1* was downregulated in BC tissues and promotes metastasis by upregulating MMP2 through *circEHMT1*/miR-1233-3p/KLF4 axis (Lu M. et al., 2020). Overexpression of *circEHMT1* inhibited migration and invasion of BC cells by reducing MMP2 expression (Lu M. et al., 2020). *Circ_0011946* functions by regulating the expression of replication factor C subunit 3 (RFC3), and silencing it suppressed migration and invasion of BC cells (Zhou J. et al., 2018). *CircGFRA1* was upregulated in TNBC cells and functions by regulating the expression of its cognate GFRA1 (GDNF Family Receptor Alpha 1) transcript by sequestering miR-34a, and its knockdown induces apoptosis (He et al., 2017). *Circ_0001982* was overexpressed in BC tissues and promotes BC tumorigenesis by sponging miR-143 (Tang et al., 2017). Silencing of *circ_0001982* suppressed cell proliferation, invasion, and induced apoptosis in BC cells (Tang et al., 2017). Interestingly, *circTADA2A-E6* and *circTADA2A-E5/E6*, originating from the TADA2A gene, were significantly downregulated in TNBC patients (Xu et al., 2019). *CircTADA2A-E6* displays tumor-suppressor properties and functions as a miR-203a-3p sponge and restores the expression of its target *SOCS3* (Xu et al., 2019). The knockdown of *circTADA2A-E6* promotes proliferation, clonogenicity, migration, and invasion in BC cells (Xu et al., 2019). *CircFBXW7* is downregulated in TNBC cell lines, it codes for a microprotein with tumor-suppressive functions in TNBC (Ye F. et al., 2019). Overexpression of *circFBXW7* suppressed cell proliferation, migration, and reversed tumor growth in TNBC cells (Ye F. et al., 2019). *CircCDYL* promoted autophagy by the miR-1275-ATG7/ULK1 axis to enhance the malignant progression of BC cells, its knockdown slows down tumorigenesis by modulating autophagy (Liang et al., 2020).

#### Hepatocellular Carcinoma (HCC)

*CDR1as* is overexpressed in HCC resulting in enhanced proliferation and invasion (Yu et al., 2016). Knockdown of *CDR1as* resulted in increased availability of miR-7 and downregulation of its target genes CCNE1 and PIK3CD, leading to inhibition of cell proliferation and invasion (Yu et al., 2016). Levels of *circMTO1* were decreased in HCC, its overexpression in HCC cells sponges oncogenic miR-9 to promote the expression of tumor-suppressor p21 resulting in reduced tumor cell proliferation, metastasis, and invasion (Han et al., 2017). Expression of *circ-10720* promotes EMT by inducing transcription factor, Twist1 (Meng et al., 2018). It promotes migration, invasion, and EMT by stabilizing mesenchymal marker vimentin by sponging miR-1246, miR-578, and miR-490-5p (Navarro, 2019). Depletion of *circ-10720* inhibited Twist1-induced metastasis (Meng et al., 2018; Navarro, 2019). Elevated *circRNA-100338* induced mTOR signaling *via* the *circRNA-100338*/miR-141-3p/RHEB axis (Huang X. Y. et al., 2020). The depletion of *circ_100338* reduced the activity of mTOR signaling pathway and suppressed HCC tumorigenesis and progression (Huang X. Y. et al., 2020). *Circ_0067934* functions by modulating the miR-1324/FZD5/Wnt/β-catenin axis to enhance migration, invasion, and proliferation of HCC cells (Zhu et al., 2018). Silencing of *circ_0067934* suppressed proliferation, migration, and invasion of HCC cells (Zhu et al., 2018). *CircSMARCA5* is downregulated in HCC tissues and inhibits proliferation, invasion, and metastasis of HCC cells by promoting the expression of the tumor-suppressor TIMP3 by sequestering miR-17-3p and miR-181b-5p (Li Z. et al., 2019). Overexpression of *circSMARCA5* inhibits the proliferation and migration of HCC cells (Li Z. et al., 2019). *CircPTGR1* promoted HCC progression *via* the miR-449a/MET pathway and its knockdown reduced HCC progression (Chen et al., 2015; Wang G. et al., 2019). *CircRHOT1* facilitated HCC progression by recruiting TIP60, a histone acetyltransferase to the nuclear orphan receptor NR2F6 promoter to enhance its expression (Wang L. et al., 2019). CRISPR/Cas9-based depletion of *circRHOT1* suppressed proliferation, migration, and invasion, and promoted apoptosis in HCC cells (Wang L. et al., 2019). *CircTRIM33-12* modulates TET1-induced DNA demethylation by sponging miR-191 (Zhang P. F. et al., 2019). Overexpression of *circTRIM33-12* inhibited proliferation and invasion of HCC cells (Zhang P. F. et al., 2019). *Circ-BIRC6* facilitates HCC progression by acting as a miR-3918 sponge and thus targeting the miR-3918/Bcl2 axis (Tang et al., 2015). Its knockdown resulted in decreased HCC cell proliferation, migration, and invasion, and enhanced apoptosis (Tang et al., 2015). *Circ_0070269* levels are downregulated in HCC tissues and it facilitates HCC progression by regulating the miR-182/NPTX1 axis (Zhang P. F. et al., 2019). Its overexpression suppresses the proliferation, and invasion of HCC cells (Zhang P. F. et al., 2019). *CircADAMTS13* was downregulated in HCC tissues (Qiu L. et al., 2019). It sequesters oncogenic miR-484, and overexpression of *circADAMTS13* resulted in a significant reduction in HCC cell proliferation (Qiu L. et al., 2019).

#### Glioblastoma (GBM)

*CircNFIX* acts as a sponge for miR-34a-5p which targets the Notch signaling pathway in GBM cells (Xu et al., 2018). The knockdown of *circNFIX* inhibited cell proliferation and migration of GBM cells by downregulating *NOTCH1* (Xu et al., 2018). CircRNA *cZNF292* is an oncogenic circRNA that promotes angiogenesis in GBM (Yang P. et al., 2019). Downregulation of *cZNF292* reduced proliferation in GBM cells and suppressed human glioma tube formation by modulating Wnt/β-catenin signaling pathway (Yang P. et al., 2019). *Circ_0037251* enhances GBM progression by sponging miR-1229-3p and upregulating mTOR (Cao et al., 2019). Knockdown of *circ_0037251* inhibited the expression of mTOR leading to increased apoptosis and promoting cell-cycle arrest (Cao et al., 2019). *CircMAPK4* functions as an oncogene to enhance GBM cell survival by sponging miR-125a-3p and regulating the p38/MAPK pathway, its downregulation induces apoptosis of GBM cells (He et al., 2020a). *Circ-U2AF1* enhanced glioma cell proliferation, migration, and invasion by sponging miR-7-5p and increasing the expression of NOVA2 (Li, 2019b). The knockdown of *circ-U2AF1* decreased the migration and invasion abilities of glioma cells by downregulating NOVA2 (Li, 2019a). *Circ_0001946* was downregulated in GBM cells and functions by sponging miR-671-5p (Li, 2019a). Overexpression of *circ_0001946* reduced the migration, invasion, and proliferation of GBM cells by inhibiting the pro-tumorigenic effects of miR-671-5p (Li, 2019a). *CircNT5E* promotes GBM tumorigenesis by sponging miR-422a and its CRISPR/Cas9-mediated deletion suppressed proliferation, migration, and invasion of GBM cells (Wang R. et al., 2018a). *Circ_0029426* facilitates tumorigenesis by sequestering miR-197, its silencing suppressed proliferation, migration, and invasion, and promoted apoptosis of GBM cells (Zhang G. et al., 2019). *Circ-TTBK2* promotes GBM malignancy by modulating the miR-217/HNF1β/Derlin-1 pathway, and its knockdown blocked GBM progression (Zheng et al., 2017). *CircMMP9* elicits its oncogenic function by sequestering miR-124 and upregulating the expression of its targets, cyclin-dependent kinase 4 (CDK4), and aurora kinase A (AURKA) (Wang R. et al., 2018b). Silencing of *circMMP9* inhibited proliferation, migration, and invasion of GBM cells (Wang R. et al., 2018b).

#### Lung Cancer (LC)

*CircRNA_103809* functions as a miR-4302 sponge leading to the ZNF121-mediated increase in MYC expression (Liu W. et al., 2018). Downregulation of *circRNA_103809* resulted in delayed tumor growth and inhibited cell proliferation and invasion in LC cells (Liu W. et al., 2018). *Circ_0020123* sequesters miR-144 and causes upregulation of ZEB1 and EZH2 which are critical for EMT and its knockdown suppresses NSCLC growth and metastasis (Qu et al., 2018). C*ircFADS2* sponges tumor-suppressor miR-498, its silencing reduced invasion and proliferation in LC cells (Zhao F. et al., 2018). *Circ_0000064* levels were elevated in LC tissues and its ablation attenuates cell proliferation and promotes cell apoptosis in LC cells (Luo et al., 2017). *CircRNA_102231* is overexpressed in LUAD tissues and its inhibition resulted in reduced cell proliferation, and invasion (Zong et al., 2018b). *Circ_0033155* is downregulated in NSCLC tissues, and its overexpression resulted in reduced cell proliferation, migration, and colony formation in NSCLC (Gu et al., 2018). *CircRNA_100876* acts as a miR-136 decoy, which targets MMP13 (Yao J. T. et al., 2017). Its silencing suppressed MMP13 expression and increased extracellular matrix formation (Yao J. T. et al., 2017). *CircPTK2* was downregulated in NSCLC cells during TGF-β induced EMT (Wang L. et al., 2018). *CircPTK2* functions as the miR-429/miR-200b3p sponge and reduced the expression of tumor-suppressor T1F1γ, consistent with this its overexpression in NSCLC cells augments T1F1γ expression and reduces TGF-β induced EMT (Wang L. et al., 2018). *CircPVT1* facilitates the increased expression of E2F2 by sponging miR-125b, and its downregulation increased apoptosis *via* E2F2 signaling pathway (Li X. et al., 2018c). Tan et al. identified the oncogenic, *f-circEA-4a* in plasma of NSCLC patients with *EML4-ALK* fusion (Tan et al., 2018). Its silencing reduced cell proliferation, metastasis, and invasion (Tan et al., 2018). The same group identified another oncogenic fusion-circRNA, *f-circEA-2a* produced from *EML4-ALK* fusion bearing an “AA” motif at the junction site. Its overexpression was reported to promote cell migration and invasion in NSCLC cells (Tan et al., 2018). Lower expression levels of *circ-FOXO3* were observed in NSCLC tissues and its overexpression reduced NSCLC development by sponging miR-155 and releasing repression of FOXO3 (Zhang Y. et al., 2018).

#### Gastric Cancer (GC)

*CDR1as* modulates PTEN/PI3K/AKT signaling pathway and confers an aggressive oncogenic phenotype to GC cells (Pan et al., 2018). Downregulation of *CDR1as* induced cell death and restricts GC progression (Li X. et al., 2019). *Circ_100269* is downregulated in GC tissues and its overexpression sponges oncogenic miR-630 suppressing GC growth (Zhang Y. et al., 2017). *Circ_104916* was downregulated in GC tissues and cell lines, its overexpression suppressed cell proliferation, migration, and EMT (Li J. et al., 2017). *CircPDSS1* sponges tumor-suppressing miR-186-5p and upregulate the oncogene NEK2 in GC tissues, and its depletion inhibited cell proliferation (Ouyang et al., 2019). *Circ_0023642* is upregulated in GC and regulates the EMT signaling pathway, and its depletion results in tumor inhibition, reduced cell proliferation, and metastasis due to the downregulation of N-cadherin, Vimentin and Snail (Zhou L. H. et al., 2018). *Circ-ATAD1* promotes GC progression by modulating the miR-140-3p/YY1/PCIF1 signaling axis (Zhang L. et al., 2020). Consistent with its oncogenic function, the depletion of *circATAD1* reduced cell viability and colony formation of GC cells (Zhang L. et al., 2020). Interestingly, *circFN1* was highly expressed in cisplatin-resistant GC tissues and promoted cisplatin-resistance by enhancing cell viability and suppressing apoptosis, by sequestering miR-182-5p (Huang X. X. et al., 2020). The knockdown of *circFN1* promotes cisplatin-sensitivity and apoptosis in GC cells (Huang X. X. et al., 2020). *CircCACTIN* promotes GC progression by sponging miR-331-3p and increasing expression of TGFBR1 (Transforming growth factor-β receptor type 1) (Zhang L. et al., 2019). Knockdown of *circCACTIN* suppressed proliferation, migration, invasion, and EMT of GC cells (Zhang L. et al., 2019). *Circ-CEP85L* is downregulated in GC tissues, it acts as miR-942-5p sponge leading to the upregulation of NFKBIA (NFKB Inhibitor Alpha) (Lu J. et al., 2020). Consistent with this overexpression of *circ-CEP85L* inhibited proliferation and invasion of GC cells (Lu J. et al., 2020).

#### Bladder Cancer (BCa)

*CircRNA-MYLK* augments proliferation, migration, tube formation of human umbilical vein epithelial cells (HUVEC) and EMT by sponging miR-29a, and stabilizing its target VEGFA in BCa cells (Zhong et al., 2017). The depletion of *circRNA-MYLK* decreased proliferation, motility, and induced apoptosis in BCa (Zhong et al., 2017). *CircACVR2A* is downregulated in BCa tissues, it sponges miR-626 to upregulate the expression of the tumor-suppressor EYA4 (Dong et al., 2019). Consistent with this overexpression of *circACVR2A* suppressed proliferation, migration, and invasion of BCa cells and metastasis (Dong et al., 2019). In contrast to CRC*, circHIPK3* is downregulated in BCa and serves as a sponge for miR-558 (Li Y. et al., 2017). It prevents angiogenesis by inhibition of heparanase (HPSE), a positive regulator of VEGF expression (Li Y. et al., 2017). Overexpression of *circHIPK3* can be used to reduce aggressiveness and metastasis in BCa cells by targeting the miR-558/heparanase axis (Li Y. et al., 2017). *CircITCH* was downregulated in BCa samples, overexpression of *circITCH* upregulates p21 and PTEN expression by sponging oncogenic miRNAs, miR-17/miR-224, leading to inhibition of BCa cell proliferation, migration, and invasion (Yang C. et al., 2018).

#### Ovarian Cancer

*Circ_0061140* is upregulated in ovarian cancer cell lines and regulates the miR-370/FOXM1 pathway by sequestering miR-370 (Chen Q. et al., 2018). Knockdown of *circ_0061140* suppressed proliferation and migration in GC cells (Chen Q. et al., 2018). *CDR1as* expression is upregulated in OC tissues and it correlated with poor prognosis for TNM stages, lymph node metastasis, and reduced OS (Luo Y. H. et al., 2020). *CDR1as* sponges miR-641 causing up-regulation of ZEB1 and MDM2 expression to promote OC (Luo Y. H. et al., 2020). A large number of circRNAs are misexpressed in primary and metastatic sites of epithelial ovarian carcinoma and their expression exhibits an inverse trend as compared to their linear counterparts in many cancer-related pathways and signaling pathways like NFkB, PI3k/AKT, and TGF-β (Ahmed et al., 2016). Accumulating evidence suggest that circRNA are associated with the initiation and progression of OC (Shabaninejad et al., 2019).

#### Osteosarcoma (OSC)

*CircUBAP2* acts miR-143 sponge and upregulates its target Bcl-2 in OSC (Zhang H. et al., 2017). Depletion of *circUBAP2* suppressed proliferation and induced apoptosis in OSC cells (Zhang H. et al., 2017). *Circ_0009910* sequesters miR-449a which targets IL6R (interleukin 6 receptor), and its knockdown induced cell-cycle arrest, inhibited proliferation and induced apoptosis is OSC cells (Deng et al., 2018). *CircPVT1* was upregulated in the OSC tissues and chemoresistant cell lines, its silencing reversed chemoresistance by decreasing the expression of ABCB1 (ATP Binding Cassette Subfamily B Member 1) (Kun-peng et al., 2018b). *Circ_001564* promotes tumorigenicity by sequestering miR-29c-3p, its depletion suppressed the proliferative activity, induced cell-cycle arrest, and promoted apoptosis (Song and Li, 2018). *Circ_0002052* was downregulated in OSC tissues and suppresses Wnt/β-catenin signaling pathway by promoting APC2 expression *via* sponging miR-1205 (Wu Z. et al., 2018). Overexpression of *circ_0002052* suppresses migration and invasion in OSC cells (Wu Z. et al., 2018). *CircNASP* functions by sponging miR-1253 leading to the upregulation of FOXF1 (Huang et al., 2018). Ablation of *circNASP* by siRNAs inhibits the proliferation, cell-cycle progression, and invasion in OSC cells (Huang et al., 2018).

### Head and Neck Squamous Cell Carcinoma (HNSCC)

#### Esophageal Squamous Cell Carcinoma (ESCC)

*Circ_0067934* was upregulated in ESCC tumor tissues and cell lines, also its silencing inhibited proliferation and migration of ESCC cells (Xia et al., 2016). *Circ_0000337* was upregulated in ESCC tissues and sequesters miR-670-5p, its depletion inhibits cell proliferation, migration, and invasion (Song et al., 2019).

#### Oral Squamous Cell Carcinoma (OSCC)

*CircUHRF1* functions as a miR-526b-5p sponge and positively regulates c-Myc, which induces TGF-β1 and ESRP1 (Epithelial Splicing Regulatory Protein 1) expression (Zhao W. et al., 2020). The knockdown of *circUHRF1* reduces migration, invasion, and EMT of OSCC cells (Zhao W. et al., 2020).

## Conclusion and Future Perspectives

CircRNAs which were considered mere splicing artifacts until a few years ago are poised to occupy a center stage in the world of regulatory RNAs. CircRNAs regulate the cellular transcriptome by diverse mechanisms and contribute to a range of cellular functions. They are involved in regulating all the major hallmarks of cancer and can serve as promising biomarkers for cancer diagnosis and prognosis. Unfortunately, so far none of these circRNAs have reached the clinics, and evaluation of a combination of circRNAs as a signature for diagnosis and correlation with clinical features is the likely way forward. CircRNAs also have immense potential for use as therapeutic targets. Novel and effective therapies can be designed by either modulating the endogenous expression circRNAs or by exogenous delivery of artificially engineered circRNAs. At present, the use of circRNAs as therapeutic agents is restricted to the bench and warrants further investigation for clinical use. The circRNA which may be suitable for therapeutic targeting, may also be different for distinct cancer types. However, the aberrant *CDR1as* expression is common to several cancer types, and targeting it for treating many different cancer types has shown promising results *in vitro* and *in vivo*. Development of RNA-based therapeutics for targeting *CDR1as* for clinical use has the potential to emerge as a single-target therapy for multiple cancers and is worth further investigation.

## Author Contributions

PK and VS conceived and designed the manuscript. AR, SB, VS, and PK wrote the manuscript. All authors read and approved the final version of the manuscript.

## Conflict of Interest

The authors declare that the research was conducted in the absence of any commercial or financial relationships that could be construed as a potential conflict of interest.

## Glossary

5′UTR, 5′Untranslated region; ABCB1, ATP Binding Cassette Subfamily B Member 1; ADAR1, Adenosine deaminase 1 acting on RNA; ALP, Alkaline phosphatase; AML, Acute myeloid leukemia; APC, Adenomatous polyposis coli; ASOs, Antisense oligonucleotides; AURKA, Aurora kinase A; BAG4, BAG cochaperone 4; BC, Breast cancer; BCa, Bladder Cancer; BCLC, Barcelona clinic liver cancer; CA-724, Carbohydrate antigens-724; CC, Cervical cancer; CCND1, Cyclin D1; CCNE1, Cyclin E1; CDK4, Cyclin dependent kinase 4; CEA, Carcinoembryonic antigen; circRNAs, Circular RNAs; CLL, Chronic lymphocytic leukemia; CML, Chronic lymphocytic leukemia; CNNM3, CBS domain divalent metal cation transport mediator 3; CRC, Colorectal cancer; CSE1L, Chromosome segregation 1 like; CXCL6, Chemokine (C-X-C Motif) ligand 6; DFS, Disease free survival; DHX9, DExH-Box Helicase 9; DLBCL, Diffuse large B-cell lymphoma; dsRBDs, Double stranded RNA-binding domains; E2F5, E2F transcription factor 5; EGFR, Epidermal growth factor receptor; EMT, Epithelial-mesenchymal transition; ER, Estrogen receptor; ESCC, Esophageal squamous cell cancer; ESRP1, Epithelial Splicing Regulatory Protein 1; EYA4, EYA transcriptional coactivator and phosphatase 4; EZH2, Enhancer of zeste homolog 2; FASN, Fatty acid synthase; FBXW7, F-Box and WD repeat domain containing 7; FIGO, Federation of gynecology and obstetrics; FLT3, Fms-like tyrosine kinase 3; FMNL2, Formin like protein 2; FOXO3, Forkhead Box O3; FZD3, Frizzled class receptor 3; GAGE1, G-antigen 1; GBM, Glioblastoma; GC, Gastric cancer; GFRA1, GDNF family receptor alpha 1; HBV, Hepatitis B virus; HCC, Hepatocellular carcinoma; HNRNPL, Heterogeneous nuclear ribonucleoprotein; HNSCC, Head and Neck Squamous cell cancer; HPGD, Hydroxyprostaglandin dehydrogenase; HPSE, Heparanase; HSCC, Hypopharyngeal squamous cell carcinoma; HuR, Hu-antigen R; HUVEC, Human umbilical vein epithelial cells; ICS, Intronic complementary sequences; IGHV, Immunoglobulin heavy-chain variable region; IL6R, interleukin 6 receptor; IRESs, Internal Ribososmal Entry Sites; KIF4A, Kinesin family member 4A; LASP1, LIM and SH3 protein 1,LC, Lung cancer; LFS, Leukemia-free survival; LIF, Leukemia inhibitory factor; LSCC, Laryngeal squamous cell carcinoma; LUAD, Lung adenocarcinoma; m6A, N6-methyladenosine; MACC1, Metastasis associated in colon cancer 1; MG, β -microglobulin; MM, Multiple myeloma; MMP13, Matrix metalloproteins 13; NF1, Neurofibromin 1; NF90, Nuclear factor 90; NF110, Nuclear factor 110; NFKBIA, NFKB Inhibitor Alpha; NPM1, Nucleophosmin; NSCLC, Non small cell lung carcinoma; ORFs, Open reading Frames; OS, Overall survival; OSC, Osteosarcoma; OSCC, Oral squamous cell carcinoma; PCK1, Phosphoenolpyruvate carboxykinase 1; PDK1, 3-phosphoinositide-dependent protein kinase; PFS, Progression-free survival; PIK3CD, Phosphotidylinositol-4,5-Bisphosphate 3-kinase catalytic subunit delta; PLK1, Polo like kinase 1; PRKACB, Protein kinase CAMP-activated catalytic subunit Beta; PTBP1, polyprimidine tract binding protein 1; QKI, Quaking; RBM20, RNA-binding motif protein 20; RBPs, RNA-binding proteins; RFC3, Replication factor C subunit 3; RFS, Recurrance free survival; RT-qPCR, Real-time quantitative polymerase chain reaction; shRNAs, short hairpin RNAs; siRNAs, Small interfering RNAs; SOCS3, Suppressor of cytokine signaling 3; T1F1γ, Transcription intermediatory factor 1-gamma; TET1, Ten-eleven translocation methyclcytosine dioxygenase 1; TERT, Telomerase reverse transcriptase; TGFBR1, Transforming growth factor-β receptor type 1; TIMP3, TIMP Metallopeptidase inhibitor 3; TMZ, temozolomide; TNBC, Triple negative breast cancer; TNKS, Tankyrase; TNM, Tumor; Node and Metastasis; U1snRNP, U1 small nuclear ribonucleoprotein particle; ULK1, Uridine kinase-like protein 1; USP28, Ubiquitin specific peptidase 28; VEGFA, Vascular endothelial growth factor A; YAP, Yes-associated protein 1; ZEB1, Zinc finger E-box binding homeobox 1.
